# Evolving Strategies to Eliminate the CD4 T Cells HIV Viral Reservoir *via* CAR T Cell Immunotherapy

**DOI:** 10.3389/fimmu.2022.873701

**Published:** 2022-04-29

**Authors:** Jarrod York, Kavitha Gowrishankar, Kenneth Micklethwaite, Sarah Palmer, Anthony L. Cunningham, Najla Nasr

**Affiliations:** ^1^ Centre for Virus Research, The Westmead Institute for Medical Research, Westmead, NSW, Australia; ^2^ Centre for Cancer Research, The Westmead Institute for Medical Research, Westmead, NSW, Australia; ^3^ Children’s Cancer Research Unit, Kids Research, The Children’s Hospital at Westmead, Sydney Children’s Hospitals Network, Westmead, NSW, Australia; ^4^ Faculty of Medicine and Health, Sydney Institute for Infectious Diseases, School of Medical Sciences, The University of Sydney, Sydney, NSW, Australia; ^5^ Blood Transplant and Cell Therapies Program, Department of Haematology, Westmead Hospital, Sydney, NSW, Australia; ^6^ NSW Health Pathology Blood Transplant and Cell Therapies Laboratory – Institute of Clinical Pathology and Medical Research (ICPMR) Westmead, Sydney, NSW, Australia

**Keywords:** HIV-1, CD4, CD8, chimeric antigen receptor, latency, reactivation

## Abstract

Although the advent of ART has significantly reduced the morbidity and mortality associated with HIV infection, the stable pool of HIV in latently infected cells requires lifelong treatment adherence, with the cessation of ART resulting in rapid reactivation of the virus and productive HIV infection. Therefore, these few cells containing replication-competent HIV, known as the latent HIV reservoir, act as the main barrier to immune clearance and HIV cure. While several strategies involving HIV silencing or its reactivation in latently infected cells for elimination by immune responses have been explored, exciting cell based immune therapies involving genetically engineered T cells expressing synthetic chimeric receptors (CAR T cells) are highly appealing and promising. CAR T cells, in contrast to endogenous cytotoxic T cells, can function independently of MHC to target HIV-infected cells, are efficacious and have demonstrated acceptable safety profiles and long-term persistence in peripheral blood. In this review, we present a comprehensive picture of the current efforts to target the HIV latent reservoir, with a focus on CAR T cell therapies. We highlight the current challenges and advances in this field, while discussing the importance of novel CAR designs in the efforts to find a HIV cure.

## Introduction

Over forty years of HIV research has resulted in the management of this infection as a chronic disease, primarily due to the advent of effective antiretroviral therapy (ART) in addition to improved community, sexual, drug and occupational health practices (1). However, despite these successes, there is currently no effective vaccine for the virus, and HIV cannot be fully eradicated from patients due to the establishment of the HIV latent reservoir, defined here as cells containing replication-competent HIV. In 2018, 1.7 million people were newly infected with HIV worldwide, bringing the global disease burden to over 37 million. Currently anti-retroviral therapy (ART) inhibits HIV replication at several stages of the virus lifecycle (2). If ART is received early during acute infection, long-term control without viral rebound following withdrawal of treatment has been observed in patients, referred to as post-treatment controllers (PTCs) (3). The immune system in PTCs is able to control viremia and limit the HIV latent reservoir size even after the cessation of ART (3). Levels of CD4 T cell activation have been found to be lower in PTCs than in non-controllers. Furthermore, CD8 T and natural killer (NK) cell responses are more efficient, and levels of inflammation are low (4). Although ART has significantly reduced the morbidity and mortality associated with HIV infection (2), the enduring pool of replication-competent proviruses in latently infected cells requires lifelong treatment adherence which is associated with long-term toxicities, issues of compliance, expense, and inconvenience. Furthermore, it has been shown that HIV-infected individuals on ART have an increased risk of malignancies, cardiovascular and neurologic disease (5). The latter is attributed to HIV infection of macrophages, astrocytes and microglia in the central nervous system (6). However, the largest HIV reservoir reside in resting CD4 T cells. We and others have previously reviewed the CD4 T cell subsets in which HIV persists (7, 8). In addition, our recent study has revealed the HIV proviral landscape is different across naïve and memory CD4 T cell subsets and that the viral protein Nef plays a role in the persistence of genetically intact HIV within the effector memory CD4 T cells (9). Reactivation of replication-competent virus (intact) in CD4 T cells upon cessation of treatment remains a major drawback of ART (10). Therefore, the latent HIV reservoir acts as the main barrier to immune clearance and HIV cure.

## Innate and Adaptive Immune Response to HIV Infection

Innate immunity is mediated upon pathogen encounter by cells to directly destroy invading pathogens or control them *via* production of inflammatory cytokines such as interferon (IFN) and interleukin (IL)-15. Adaptive immunity can be humoral acting in extracellular spaces *via* antibody production by B cells, or T cell-mediated effectors which act once pathogens invade cells leading to the induction of memory CD4 and CD8 T cells for faster responses during pathogen re-exposure.

### Interferon and the Innate Immune Response

Type I IFNs include IFNα, IFNβ, IFNϵ, IFNω, and IFNκ (11). Thirteen subtypes of human IFNα and IFNβ signal through the IFNα receptor (IFNAR) (12). Type II and III IFNs include IFNγ and IFNλ1-4, which signal through the IFNγ and IFNλ receptors, respectively (11). Type I and type II IFNs are key mediators of antiviral immunity, whilst type III IFN activity is limited to epithelial cell surfaces due to restricted expression of the IFN-λ receptors (13). We have previously reviewed the induction of IFN in myeloid DCs, macrophages, CD4 T cells and pDCs (14) and outlined that many viruses interfere with IFN induction to evade innate immune recognition. HIV is no exception as we have shown that HIV inhibits IFNβ production in DCs and macrophages (15) *via* the HIV-accessory protein Vpr which blocks the phosphorylation of TBK1, preventing the phosphorylation of IRF3 and its subsequent translocation to the nucleus to induce IFNβ (16). In CD4 T cells, Vpu, Vpr or Vif directly degrade IRF3 (17). Despite HIV mediated blocking of IFNβ production in its key target cells, IFN has been detected in the circulation of HIV patients within 1-2 weeks of infection (18) with pDCs being the main source of IFN production. Therefore, pDCs can compensate for the loss of IFN inhibition in HIV key target cells. pDCs and IFN and have been associated with antiviral responses that limit early SIV/HIV replication and dissemination in macaque and humanized mouse models respectively (19, 20). Moreover, Type I IFNs enhance immune cell activation and effector functions, particularly the proliferation and survival of NK cells, DC maturation, priming of T cell responses, promotion of Th cell survival, activation and expansion of CD8 T cell, B cell class switching and affinity maturation (21).

### Natural Killer Cells and the Innate Immune Response

To control initial HIV infection, NK cells are activated and proliferate prior to peak viremia (22). NK cell immunoglobulin-like receptors can interact with cells expressing human leukocyte antigen (HLA) molecules to limit HIV viral replication during acute infection. However, similar to the inhibition of type I IFN responses in early HIV infection, HIV has evolved mechanisms to decrease expression of ligands important in triggering NK cell cytotoxic responses and therefore interferes with lysis of infected cells (23).

### Adaptive Immune Responses

Adaptive immune responses are initiated as innate immunity involving the NK cytotoxic response and IFN induction is inhibited. However, there is a dampened CD4 T cell response in acute HIV infection due to their significant early depletion upon exposure to HIV (24). Early ART administration to control viremia prevents the killing of CD4 T cells and rescues potent CD4 T cell responses (25). The initial adaptive immune response depends mainly on cytotoxic CD8 T cells to limit or inhibit viral spread. However, a rapid decline of CD8 T cell responses occurs limiting its effectiveness. The recapitulation of this phenomenon in a CD4 depleted mouse model suggests that CD4 T cells are required for the maintenance of long-lived memory CD8 T cells in the context of acute HIV infection (26).

Although the early CD8 T cell response peaks later than viral load, there is much evidence for the importance of CD8 T cells in controlling HIV replication *via*: the association of HIV-specific CD8 T cells with a decrease in HIV-infected cells in acutely infected patients (27); restriction of viral replication and disease progression in chronically infected patients, especially elite controllers (28); prevention of viral escape mutations (29); maintaining viral suppression in SIV-infected macaques (30). Various studies in macaques have shown that stimulation of SIV-specific CD8 T cell responses by vaccines can attenuate SIV infection (31) and *in vivo* exhaustion of CD8 T cells in this model limited viral control in acute infection (32). CD8 T cell responses during initial HIV infection target mutable regions of the virus to decrease replication efficiency and contribute to the control of HIV infection (33). This early CD8 T cell response is observed days prior to the peak of viremia and targets epitopes found in HIV Env and Nef (34). However, it has been demonstrated that escape mutants, which cannot be recognized by initially generated cytotoxic CD8 T cells, are generated within 10 to 21 days post infection in response to potent T cell responses (35). Therefore, although the initial CD8 T cell response restricts viremia in acute HIV infection, their target epitopes are subsequently mutated resulting in immune escape.

In addition, HIV-specific CD8 T cell cytotoxic responses decrease with disease progression, due to a reduction in their frequency, increased activation, and immune exhaustion (36). CD8 T cells with higher expression of activation and immune exhaustion markers such as programmed death (PD)-1 and T-cell immunoglobulin and mucin-domain containing-3 (TIM-3) have been detected in untreated HIV-infected patients in contrast to seronegative patients (37). Expression of PD-1 is associated with a lower degranulation capacity and a diminished population of IL-17-producing cells following polyclonal stimulation (38). Exhausted CD8 T cells are also more susceptible to apoptosis (39). The loss of CD4 T cell assistance, persistent viral antigen load, chronic inflammation (40) and lack of co-stimulation (41) have been proposed to contribute to CD8 T cell dysfunction during chronic HIV infection. Furthermore, increased expression of inhibitory molecules, competitive signalling between TCRs, upregulated inhibitory receptors on the responding CD8 T cells and the modulation of intracellular signalling pathways can all contribute to the lack of co-stimulation and dampening of the CD8 T cell response to infection (42).

## Strategies to Eliminate the HIV Latent Reservoir

Current ART therapies targeting HIV suppress viral replication to undetectable levels allowing infected individuals to live without clinical symptoms, provided ART is maintained (43). Therefore, development of a cure to HIV patients relies on approaches aimed at eliminating cells harbouring latent and replication competent virions or removing the HIV provirus from the genome by gene editing. Some of these approaches have been addressed recently by Deeks et al. and are summarised below (8).

### Block and Lock

Several compounds have been developed to inhibit both the basal and signal induced activity of the nuclear transcription factor NFκB, a key mediator of HIV gene expression (44). However, the HIV LTR is sensitive to a variety of activating factors and inhibiting a single mediator such as NFκB is unlikely to silence all HIV viral RNA expression. To this end, constitutive suppression of the HIV LTR transcription using short hairpin RNAs targeting the LTR enhancer region of HIV (45) were effective in preventing viral RNA transcription. Such strategies involving recombinant proteins or RNA interference require specific targeting and expression cassettes for delivery to latently infected cells. Heat shock protein 90 (HSP90) inhibitors have also been explored as a block and lock strategy. Since heat shock proteins are required for the production of viral proteins (46), HSP90 inhibitors have been shown to suppress HIV transcription and replication (47). Perhaps the most advanced block and lock strategy employs didehydro-cortistatin A (dCA), a Tat inhibitor, to silence HIV transcription. It is known that viral Tat recruits and activates RNAPII for stimulation of HIV transcriptional elongation (48). Using dCA, Kessing et al. showed that prior treatment with dCA delayed and reduced viral rebound both *in vitro* and *in vivo* (49). In addition to silencing strategies, genome editing with CRISPR/Cas9, ZFNs and TALENs have been applied to highly conserved sequences within the LTR to eliminate the HIV provirus genome *in vitro* (50). These approaches would allow for highly specific targeting of latently infected cells, however off-target endonuclease activity may occur, and development of effective gene delivery vehicles remain important challenges.

### Kick and Kill

Latently infected resting memory T cells do not express HIV peptides on their cell surface and consequently evade immune detection and cell lysis. Therefore, HIV activation and infected cell elimination strategies, termed “kick and kill”, are based on reactivation of HIV provirus from latently infected cells for expression of viral peptides followed by elimination *via* viral cytopathic effects or CD8 T cell mediated immune responses (51). The first generation of LRAs, including histone deacetylase inhibitors (HDACi) and protein kinase C (PKC) activators were disappointing due mainly to a lack of potency and/or unacceptable toxicity (52). However, other studies have generated positive results both *in vitro* and using animal models for HIV latency, demonstrating induction of robust HIV expression using bryostatin, a PKC activator (53), and second mitochondria-derived activator of caspase (SMAC) mimetics to activate NF-kB signalling and reverse HIV-1 latency (54). Clinical trials have highlighted that a high concentration of LRAs is required for potent reactivation of latently infected cells, but this led to off-target effects and cytotoxicity (55). Lowering the toxicity of LRAs combined with multiple dosing were effective in improving the ‘kick’ strategies (55). One negative feature of LRAs is their suppressive effects on the cytolytic function of CTLs (56). Therefore, alternative strategies have been proposed, including use of either the SMAC mimetics that activate the non-canonical nuclear factor kB (NFkB) pathway (54) or the toll-like receptor 7 (TLR-7) agonists that mediate activation of HIV-specific CTLs (57). These strategies have shown increased reactivation and cell killing of latently infected cells, indicating that a robust immune response competent enough to remove the reactivated viral reservoir is needed (58).

Recently, a novel finding of HIV reactivation *via* IFN was reported (59) and showed that IFNα but no other IFN subtypes was able to efficiently reverse latency in both an *in vitro* model and in CD4 T cells collected from HIV patients on suppressive ART (59). This is similar to our recent findings of latency reversal in *in vitro* infected resting memory CD4 T cells (60) treated with IFNα8 or co-cultured with pDCs that secreted IFNα upon HIV exposure. Data from *in vitro* and humanized mice studies showed that IFNα8 and IFNα14 are more efficient at inhibiting HIV viral replication than IFNα2 (61) due to their higher affinities for the IFN receptor and the increased induction of antiviral proteins. Therefore, HIV reactivation by a specific type of IFNα should provide another promising strategy to reactivate and purge latently infected cells with the added benefit of inhibiting viral spread to adjacent cells (60) while avoiding the suppressive effects of LRAs on the cytolytic function of CD8 T cells. Altogether, this suggests that a combination of the above strategies may be required for an optimal “kick and kill” strategy.

Activation and elimination components are influenced by several factors. As HIV integrates within transcribed regions of the chromosome, it has been demonstrated that responsiveness of HIV provirus to T cell signalling and chromatin modifying agonists dependent on the site of integration (62). Therefore, a major challenge in developing robust LRAs is the broad range of responses of the integrated proviruses at distinct chromosomal locations (62). For example, although most provirus is responsive to T cell signalling induced by a combination of phorbol 12-myristate 13-acetate (PMA) and ionomycin, it has been shown that only small proportion of provirus is induced by treating patients with chromatin modifying agents (63). Due to regulation of viral transcription by cell signalling pathways linked to TCR engagement and T cell activation responses, forced induction of viral expression without promotion of mass T cell activation, leading to cytokine release syndrome, remains a major challenge (64).

As described earlier increased PD-1 and TIM-3 expression on CD8 T cells in HIV patients results in lower degranulation capacity, indicating that the immune system after ART is incapable of a sufficient anti-HIV cytotoxic T lymphocyte response to eliminate reactivated cells (65). To combat this, various strategies to augment anti-HIV cellular immune responses are being explored. IL-15 treatment has been shown *ex vivo* to enhance elimination of resting CD4 T cells isolated from HIV-patients treated with LRAs by inducing NK activity (66). B cell lymphoma 2 (Bcl-2) regulates apoptosis, inhibits T-cell mediated cytotoxicity pathways and has been identified in primary CD4 T cells that survived co-culture with HIV-specific CTLs (67). Addition of Bcl-2 antagonists in co-cultures of latently infected CD4 and CTLs reduced latently infected target cells when compared with CTL and LRAs alone (68). Adoptive cell therapies have revolutionised the way we can empower the immune response and direct it against specific targets in a precise and controlled manner. In recent years major advances have been made in engineering human T cells by introducing chimeric antigen receptors (CARs) to enable specific lysis. This approach has been approved as treatment for haematological malignancies and it is being extended to other diseases, including autoimmunity, fungal and viral infections.

### Broadly Neutralising Antibodies

Although the initial production of antibodies in response to HIV is non-neutralizing, in part due to targeting antigens on the HIV envelope transmembrane gp41, potent broadly neutralising antibodies (bnAbs) specific for conserved regions of HIV gp120 are generated in 1-2% of patients many years after initial infection (69). The mechanism by which bnAbs are generated in chronic patients is not well understood. However, recent structural, virologic and immunological studies have provided strong evidence of how virus–antibody co-evolution throughout the natural course of the disease are synergistically associated with sequential development of potent bnAbs via somatic hypermutations and maturation of antibody genes (70). Early studies employing passive immunotherapies of such anti-HIV antibodies have demonstrated improved clinical outcomes associated with reduction in plasma viral RNA load and delayed appearance of AIDS-defining illnesses (71). A limited number of studies has demonstrated that bnAbs may act on latently infected cells after their reactivation. For example, 3BNC117 induced cytolysis of latently infected cells and PGT121 significantly reduced HIV DNA after passive administration to non-human primates (72). bnAbs can bind and neutralise viral envelopes expressed on infected cells, following viral reactivation. They can enhance lysis of infected cells by antigen-dependent cellular cytotoxicity (ADCC) with the recruitment of NK cells and can also prevent seeding of additional latently infected cells (73). However, although bnAbs exhibit remarkable breadth and potency in their ability to neutralize HIV and prevent spread, they rely on functional NK cells to directly recognise and lyse infected cells during chronic HIV infection (74). In addition, latently infected cells express only integrated HIV DNA and thus remain invisible to the NK and CD8 T cells without HIV reactivation by latency reversal agents (LRAs). Currently, bnAbs are under investigation for treatment and prevention of HIV infection (75) and have demonstrated pre-clinical success in use as novel immunotherapies targeting the virus (76).

## Chimeric Antigen Receptor T Cells

**Figure 1:**
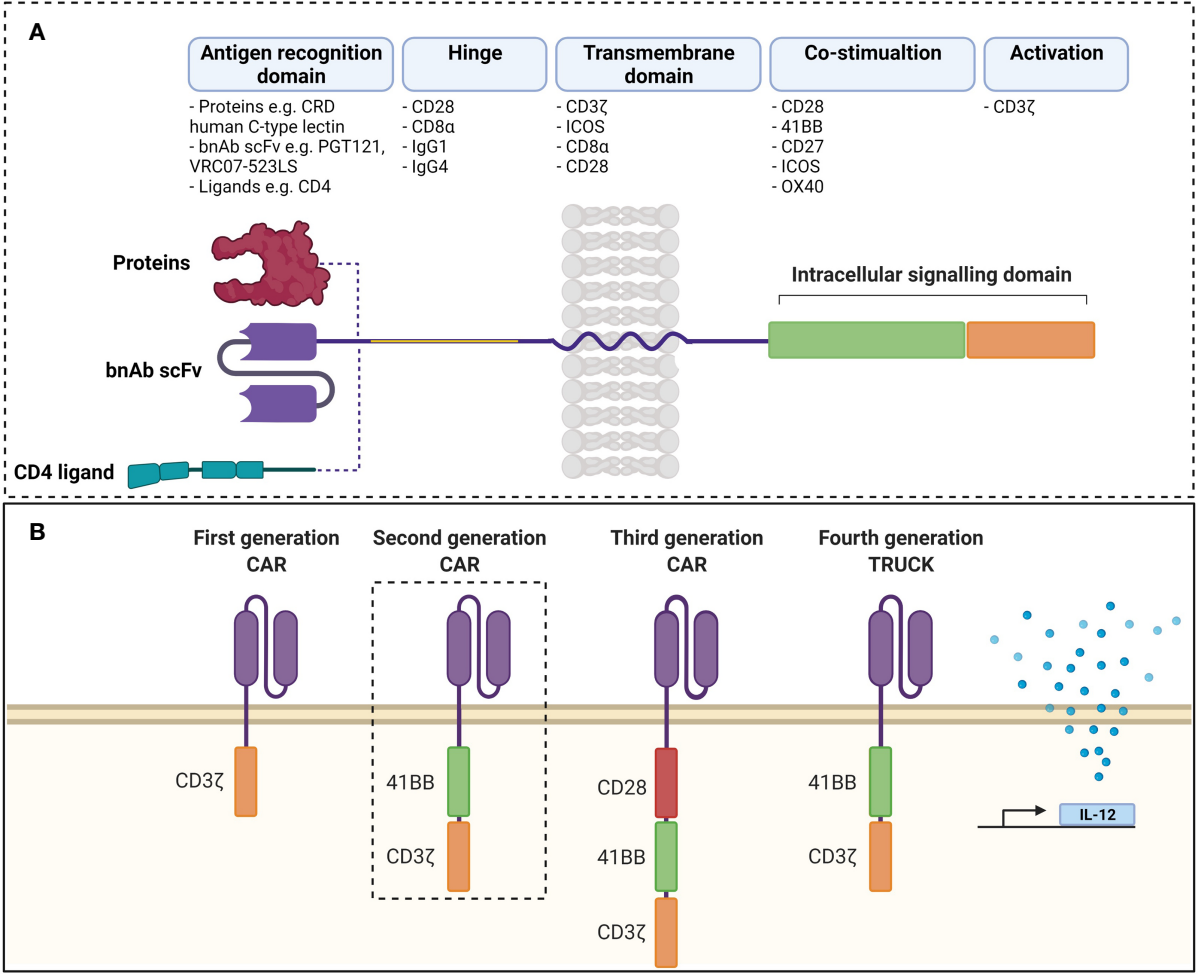
**(A)** Structure of chimeric antigen receptor. Antigen receptor domains are linked to transmembrane domains and intracellular signalling domains consisting of co-stimulatory domains and CD3ζ by hinge regions. scFvs are common as antigen recognition domains, although ligands can be used to take advantage of ligand-receptor interactions. **(B)** Evolution of chimeric antigen receptors. Antigen recognition domains are linked to CD3ζ in first generation CARs and activity was improved by addition of co-stimulatory domains in second and third generations. Fourth generation TRUCKs include inducible transcription of transgenes (such as IL-12) to second generation CARs. scFv, single chain variable fragment; CAR, chimeric antigen receptor TRUCK, T cell redirected for universal cytokine killing; bnAb, broadly neutralising antibody; CRD, carbohydrate recognition domain. Created with BioRender. Adapted from Rafiq et al. (78).

Chimeric antigen receptors (CARs) are synthetic receptors expressed on T cells that confer novel specificity by combining chosen antigen binding moieties and T cell activating functional domains. CAR expressing T cells (CAR T cells) recognise antigens independent of major histocompatibility class (MHC) presentation and can therefore be used in immunotherapy to recognise, target, and destroy antigen expressing cells in a specific manner. This MHC independent mechanism of action can be particularly attractive in targeting HIV infected cells as the virus is well known to down regulate MHC-I molecules (77). Chimeric antigen receptors have been well described by Rafiq et al. and are structured as discrete modules (**Figure 1A**) with an antigen recognition domain typically derived from single chain fragments of the variable regions (scFv) of the light and heavy chains of a monoclonal antibody (mAb) (78). In addition, novel non-antibody-based approaches have been employed which can include natural receptors, ligands, nanobodies, cytokines and peptides (78). A hinge or spacer region links the antigen recognition domain to the transmembrane domain and a T cell isignalling domain (CD3ζ domain from the TCR complex) (78). Incorporation of costimulatory domains provide the required co-stimulation for a robust response. Domains from proteins of the CD28 family (CD28 and inducible T cell co-stimulator (ICOS)), or the tumour necrosis factor receptor (TNFR) family (41BB (CD137)) and TNFR superfamily (member 4 (OX40) or CD27) have been used for this co-stimulation (78). Although several costimulatory domains have been trialled *in vitro*, CD28 and 4-1BB have been the most successful and are used in clinical products (78). For a comprehensive review of CAR engineering strategies refer to Rafiq et al. (78). Various vectors and trategies have been utilized in the production of CAR-T cells and are summarised in **Table 1**.

**Table 1:** Current *ex vivo* genetic engineering strategies for CAR expression.

Genetic engineering	Transgene delivery/Promoter	Transgene insertion/gene expression	Strengths	Limitations
Retrovirus: Lentivirus, gamma retrovirus (79)	Transduction/Exogenous	Non-targeted integration/Stable	high transduction efficiency	size restriction, expensive production, induction of an immune response, insertional mutagenesis
Transposase enzyme: PiggyBac, and sleeping Beauty (80)	Electroporation/Exogenous	Non-targeted integration/Stable	integrating larger transgenes, inexpensive production	Less developed technology than retroviral vectors, off-target cleavage and insertional mutagenesis
mRNA (81)	Electroporation/NA	Transient	Limits off-target effects	expression is rapidly diluted during T cell expansion
Non-integrative lentivirus: NILV-S/MAR (82)	Transduction/Exogenous	Episomal/Transient	Episomal maintenance prevents insertional mutagenesis	Expensive production, constant redosing required
Endonuclease enzymes: ZFNs, TALENs, CRISPR/Cas9 (83)	Electroporation/Endogenous	Targeted integration/Stable	Site-directed insertion	Not yet fully optimized

non-integrative lentivirus containing scaffold/matrix attachment region (NILV-S/MAR); zinc-finger nucleases (ZFNs); transcription activator-like effector nucleases (TALENS); clustered regularly interspaced short palindromic repeat (CRISPR).

CAR T cells have shown phenomenal success against hematologic malignancies with close to 90% response in B-cell acute lymphoblastic leukaemia and lymphoma targeting CD19 (84). A recent reportshowed persistence of CD19 CAR T cells 10 years after infusion and leading to complete remission (85), further validating CAR T cells as paradigm shifting therapies. Four CAR T cell products (targeting CD19) for leukaemia and lymphomas and one for myeloma (targeting BCMA) have been approved by the FDA as therapy. The key challenges of CAR therapy include managing adverse events like life threatening cytokine release storm (CRS) and neurotoxicity (86). CRS involves the release of effector cytokines like IL-6, IFNγ and TNFα and can be accompanied by fever, endothelial activation and vascular instability. Neurotoxicity can range from mild headache to more severe delirium, cerebral oedema or intracranial haemmorhage. Inclusion of safety switches can potentially prevent organ damage and fatality and have become an essential component of current CAR designs (78).

### Engineering CAR – T Cells to Target the HIV Reservoir

CAR T cells for HIV have been trialled since the 90s. Some have demonstrated long term persistence in peripheral blood (87–89) and have been found to traffic to areas of the HIV reservoir including the central nervous system and peripheral lymphoid tissue (90). As such, CAR T cells have the potential to revolutionise immunotherapy for HIV, when appropriate design and safety strategies are incorporated.

#### First Generation

The first-generation anti-HIV CAR-T cells linked singular CD3ζ intracellular signalling domains to antigen recognition domains directed against HIV Env using either CD4 or scFvs derived from anti-HIV Env bNAbs (**Figure 1B**). These CAR-T cells were very specific in recognizing and lysing cells expressing HIV Env *in vitro* (**Table 2A**). Using a CD8 cell line (WH3) expressing CD4-CD3ζ CARs, Romeo and Seed demonstrated that after binding to gp120 expressing HeLa cells, CD4-CD3ζ CAR-T cells exhibited extremely high cytolytic activities by chromium-release assay (91). These data were replicated in human PBMC derived CD8 T cells expressing CD4 or scFv derived from anti-HIV mAb 98.6 -CD3ζ based CARs (92). After binding to HIV Env, these CAR-T cells exhibited extremely high cytolytic activities by JAM assay, a cell lysis assay which measures the DNA retained by living cells rather than the cellular components lost by dying cells, against the CEM cell line infected with the HIV-1 IIIB strain and HEK293 expressing HIV env. Furthermore, using Jurkat cells expressing CD4 or scFv derived from anti-HIV mAb 98.6 -CD3ζ based CARs, Roberts et al. demonstrated that HEK293 env target cells stimulated Jurkat CAR-T cells to secrete IL-2 at high levels (92). Subsequently, Yang et al. confirmed that primary CD8 T cells expressing CD4 ligand or scFv derived from anti-HIV mAb 98.6 -CD3ζ based CARs lysed HIV-1 IIIB infected T1 and H9-B14 cells and inhibited HIV-1 replication *in vitro* (93). The kinetics of lysis and efficiency of inhibition were comparable to that of naturally occurring HIV-1-specific CTL clones isolated from infected individuals (93). Such preclinical studies provided clear evidence that first generation CD4 or scFv derived from anti-HIV mAb 98.6 -CD3ζ based CAR T cells exhibited significant anti-HIV activity *in vitro*.

**Table 2:** Preclinical and clinical testing of first-generation anti-HIV CAR-T cells.

Design of CAR	Outcome
*A. Preclinical studies*	
- CD8 cell line (WH3) expressing CD4 binding site-ζCAR (91).	- CD8 exhibited extremely high cytolytic activities against gp120 expressing HeLa cells by chromium-release assay
- Both Jurkat cells and primary CD8 T cells expressed CD4 binding site or scFv derived from anti-HIV mAb 98.6 -CD3ζ (92).	- CD8 exhibited extremely high cytolytic activities toward CEM and HEK293 cells expressing HIV env by JAM assay. - HEK293 expressing HIV env activated the CAR expressing Jurkat cells to secrete IL-2 at high levels.
- Primary CD8 T cells expressing CD4 ligand or scFv derived from anti-HIV mAb 98.6 -CD3ζ (93).	- Exhibited lysis of HIV-1 IIIB infected T1 and H9-B14 cells by chromium-release assay
*B. Clinical studies*	
- Phase II randomized trial: Single-dose administration of autologous CD4 and CD8 CAR-T cells both expressing CD4-CD3ζ with or without IL-2 to HIV- infected patients (n=24) with a detectable level of virus (1000 copies/mL) (88).	- CAR expression detected in 1% - 3% of circulating T cells at 8 weeks and 0.1% at 1 year - Survival of CAR was not enhanced by IL-2 - CAR DNA was observed in biopsies of bulk rectal tissue and lamina propria in 2 of 3 patients at 1 year - No significant reduction in HIV RNA or DNA
- Single and multiple infusions of autologous CD4-CD3ζ CD8 CAR-T cells with or without CD4-CD3ζ CD4 CAR-T cells between identical twins (n=33) serodifferent for HIV infection (87).	- Greater persistence for at least one year compared to 8 weeks following multiple infusions of CD8 CAR-T cells co-administered with CD4 CAR-T cells. - CAR T cells detected in lymphoid tissue biopsies at 1 year - Multiple infusions were well tolerated without substantive immunologic or virologic changes - Reduction in mean HIV RNA levels observed in patients who received repeated infusions of CD8 CAR T cells
- Phase II randomized trial: Three infusions of autologous CD4-CD3ζ CD4 and CD8 CAR-T cells compared to unmodified T cells in HIV-infected individuals (n=40) on ART with low plasma viral loads (<50 copies/mL) (89).	- Decline from baseline in HIV after CAR T cell treatment, but no meaningful difference in HIV burden was observed between treatment and placebo groups - Increase of CD4 T cell count following therapy

First generation CD4 ligand-based CAR-T cells also demonstrated stable engraftment and long-term safety in the clinical setting (**Table 2B**). However, despite these observations and the previously demonstrated anti-HIV activity *in vitro*, the CD4-CD3ζ CAR-T cells were not capable of reducing viral burden permanently in most clinical studies (87, 88). Specifically, Mitsuyasu et al. noted CAR expression in 1% - 3% of circulating T cells at 8 weeks and 0.1% at 1 year following administration of autologous CD4 and CD8 T cells expressing CD4-CD3ζ CARs with or without IL-2 to HIV-infected patients (n=24) with a detectable level of virus (1000 copies/mL). However, no significant reduction in HIV RNA or DNA was observed (88). Moreover, administration of exogenous IL-2 with CAR T cells was not shown to enhance survival. Another study showed greater persistence of CD8 T cells expressing CD4-CD3ζ CARs at 1 year after multiple infusions when co-administered with CD4 T cells expressing CD4-CD3ζ CARs compared to CD8 CAR-T cells infused without CD4 CAR-T cells in identical twins (n=33) sero-different for HIV infection (87). Furthermore, a reduction in mean HIV RNA levels was observed in patients who received repeated infusions of CD8 CAR T cells (87). Subsequently, it has been demonstrated that infusion of CD4-CD3ζ expressing CD4 and CD8 T cells led to a decline from baseline in HIV after CAR T cell treatment, but no meaningful difference in HIV burden was observed between treatment and placebo groups (89). Notably, both groups experienced a treatment-related increase in CD4 T-cell counts (89).

The failure of these clinical trials to generate robust cell lysis of their targets may be attributed to several factors: (1) low transduction efficiency and/or dose of CD4-CD3ζ CAR-T cells leading to lower efficacy (94); (2) T cell exhaustion during *ex vivo* CD4-CD3ζ CAR-T cell expansion by excessive IL-2 stimulation (95); (3) CAR design leading to low expansion rate and effector functions (96); (4) Initial ART administration prior to CD4-CD3ζ CAR T cell infusion may block the efficacy of CAR-T cells by reducing HIV Env antigen required for optimal expansion of a CD4-CD3ζ CAR-T cells *in vivo* and the production of a robust anti-HIV response (97); (5) CD4-CD3ζ CAR T cells may induce selective pressure on HIV Env to select for escape mutants (98); however, (6) the most common explanation for the failure of the CD4-CD3ζ CAR T cell clinical trials is the inclusion of CD4 as the antigen recognition domain, rendering the CD8 T cells expressing CD4 susceptible to HIV infection (97). Overall, the application of first-generation anti-HIV CAR T cell therapy in HIV patients has demonstrated a lack of adverse effects, and the generation of compartmental immunity to HIV (87–89). However, the lack of efficacy of these CAR T cells *in vivo* despite previously demonstrated significant anti-HIV activity *in vitro* led to the development of second and third generation CARs incorporating the signals require for full T-cell activation within the CAR structure to enhance their function.

#### Second Generation

Based on the success of second generation anti-CD19 CAR-T cells for hematologic malignancies, various laboratories have developed second generation anti-HIV CAR T cells and analysed them in preclinical studies (**Figure 1B**). Landmark studies have been summarised in **Table 3**. Second-generation CD8 CAR T cells incorporating the CD4 ligand or scFv derived from anti-HIV bnAbs as antigen recognition domains co-stimulated by 41BB were directly compared with first generation CARs and shown to be at least 50-fold more potent at suppressing HIV replication *in vitro* (94). Furthermore, second generation CARs demonstrated superior antigen dependent expansion, CD4 T cell protection *via* reduced cell loss *in vivo* when compared to HIV-specific CARs without co-stimulatory molecules (101). In the same study, a direct comparison of 41BB and CD28 co-stimulatory domains showed that 41BB costimulatory domains promote antigen independent T cell persistence *in vivo* (101) and that they are superior to the CD28 domain in reducing viral rebound following cessation of ART treatment. This is in accordance with other 4-1BB co-stimulatory domain containing CARs showing long term persistence (96). Primary CD4 and CD8 T cells expressing scFv derived from anti-HIV bnAbs including PGT128, PGT145, VRC07-523, or 10E8 in second generation CARs with 41BB co-stimulatory domains were developed and compared (99). All CARs incorporating scFv from each bnAb were shown to be efficacious as anti-HIV designs using HIV infected cell lines and Env transfected cells (99). However, PGT145- and VRC07-523-CARs showed the most consistent potency in cytotoxic activity (99). Perhaps most importantly, this study demonstrated the success of homology-directed recombination of CAR transgene into the CCR5 (HIV co-receptor) locus to suppress entry of HIV into these cells (99). By showing the feasibility of CAR integration to disrupt CCR5 in T cells, Hale et al. demonstrated an important consideration when developing a CAR T cell immunotherapy for HIV. The efficacy of CAR expressing CD4 T cells in the context of HIV has also been demonstrated in other studies. Focussing on HIV-resistant CD4 CAR-T cells modified by introduction of a single Asp mutation (D97N) and expressing co-stimulatory domains derived from 41BB, CD28, CD27, OX40 or ICOS, Maldini et al. showed direct elimination of env expressing cell lines *in vitro.* They also demonstrated improved proliferation and persistence of CD4 ligand based CAR expressing CD8 T cells, especially when co-injected with CD4 ligand based CAR expressing CD4 T cells *in vivo* (101). Furthermore, HIV-resistant CAR expressing CD4 T cells containing costimulatory domains from the CD28 receptor family exhibited the greatest production of effector cytokines *in vitro*. whilst 41BB co-stimulated CAR expressing CD4 T cells showed superior expansion and reduced HIV pathogenesis *in vivo* using a humanized mouse model of HIV infection (100).

**Table 3:** Recent preclinical testing of second-generation anti -HIV CAR T cells.

Design	Outcome
- Primary CD8 T cells expressing CD4 or scFv derived from anti-HIV bnAbs (VRC01, 3BNC60, PG9, PGT128, PGDM1400) based second generation CARs with 41BB or CD28 co-stimulatory domains (94).	- Had 50-fold increase in potency at suppressing HIV replication *in vitro* than first generation CAR T cells by chromium-release assay. - Superior *in vivo* expansion in response to antigen, Reduced CD4 T cell loss compared to first generation CARs. - Incorporation of 41BB costimulatory domains was superior to CD28 domains in reducing viral rebound after ART treatment and promoting T cell persistence *in vivo*
- Primary CD4 and CD8 T cells expressing scFv derived from anti-HIV bnAbs (PGT128, PGT145, VRC07-523, 10E8) second generation CARs with 41BB as a co-stimulatory domain (99). - AAV6 mediated homology-directed recombination of the CAR gene into the CCR5 locus (99).	- PGT145- and VRC07-523-CARs had consistent potency in killing of HIV infected cell lines and Env transfected cells - CCR5-edited CAR T cells more effectively suppressed viral replication
- Primary CD4 and CD8 T cells expressing CD4 ligand based second generation CARs and incorporating co-stimulatory domains derived from 41BB, CD28, CD27, OX40 and ICOS (100). - CAR modified CD4 T cells rendered HIV-resistant by introduction of a single Asp mutation (D97N) of CXCR4 (100).	- CAR T cells with CD28 costimulatory domains exhibited the greatest production of IL-2, TNF, IFNγ, (MIP)-1b and GMCSF when co-cultured with env expressing cell lines *in vitro.* - CAR T cells with 4-1BB-costimulatory domains directly eliminated env expressing cell lines *in vitro and* exhibited profound expansion. - CD4 CAR T Cells had improved proliferation and persistence of CD8 CAR T cells *in vivo*.

These data demonstrate that co-stimulatory domains along with helper functions supplied from CAR expressing CD4 T cells differentially enhance HIV-specific CAR expressing CD8 T cell function *in vivo.* Future studies may benefit from adopting strategies to develop HIV-resistant CAR expressing CD4 T cells and preferentially incorporating 41BB co-stimulatory domains.

#### Third and Fourth Generation

Third-generation HIV-specific CARs (**Figure 1B**) have not been extensively studied in HIV. A single study combining multiple intracellular signalling domains from 41BB, CD28 and CD3ζ linked to the scFv of anti-HIV bnAb VRC01 displayed increased potency in lysing env expressing cell lines *in vitro* compared to HIV recognition *via* CD4-based third generation CAR (102). Unfortunately, this study failed to directly compare the potency of their third generation CAR to an appropriately designed second generation CAR counterpart. However, an important finding of the study was that the VRC01-based third generation CAR-T cell effectively eliminated LRA-reactivated HIV-1-infected CD4 T cells isolated from infected individuals receiving ART, thus validating the development of CAR-T cell immunotherapies against latent HIV (102). The fourth generation of CAR-T cells are known as T cells redirected for universal cytokine-mediated killing (TRUCKs) and have yet to be employed in the context of HIV infection (**Figure 1B**). TRUCKs can utilise the additional secretion of IL-12 or other inflammatory mediators to attract innate immune cells and eliminate antigen negative cancer cells. CAR designs are constantly evolving with novel features being added to provide multiple functions, especially to overcome the challenges of the tissue microenvironment and future anti-HIV CARs will no doubt incorporate such features.

### Advances in Developing CAR-T Cell Therapy for HIV Cure

Successful immunotherapies for chronic viral infections, such as HIV, would need to overcome challenges of immune escape, off-target effects, and navigate immunoregulatory factors in the tissue microenvironment for a sustained and effective response. The versatile and modular nature of CARs and advances in molecular and synthetic biology thus allows for the development of a myriad of designs for these purposes to increase the efficacy and precision of anti-HIV cell therapies. Prompted by novel innovations in cancer immunotherapy, various research groups have begun designing and testing CARs to overcome shared challenges between cancer and chronic viral infections as well as HIV specific hurdles including CAR – T cell expansion and persistence, and their susceptibility to HIV infection.

#### Overcoming Immune Escape

**Figure 2:**
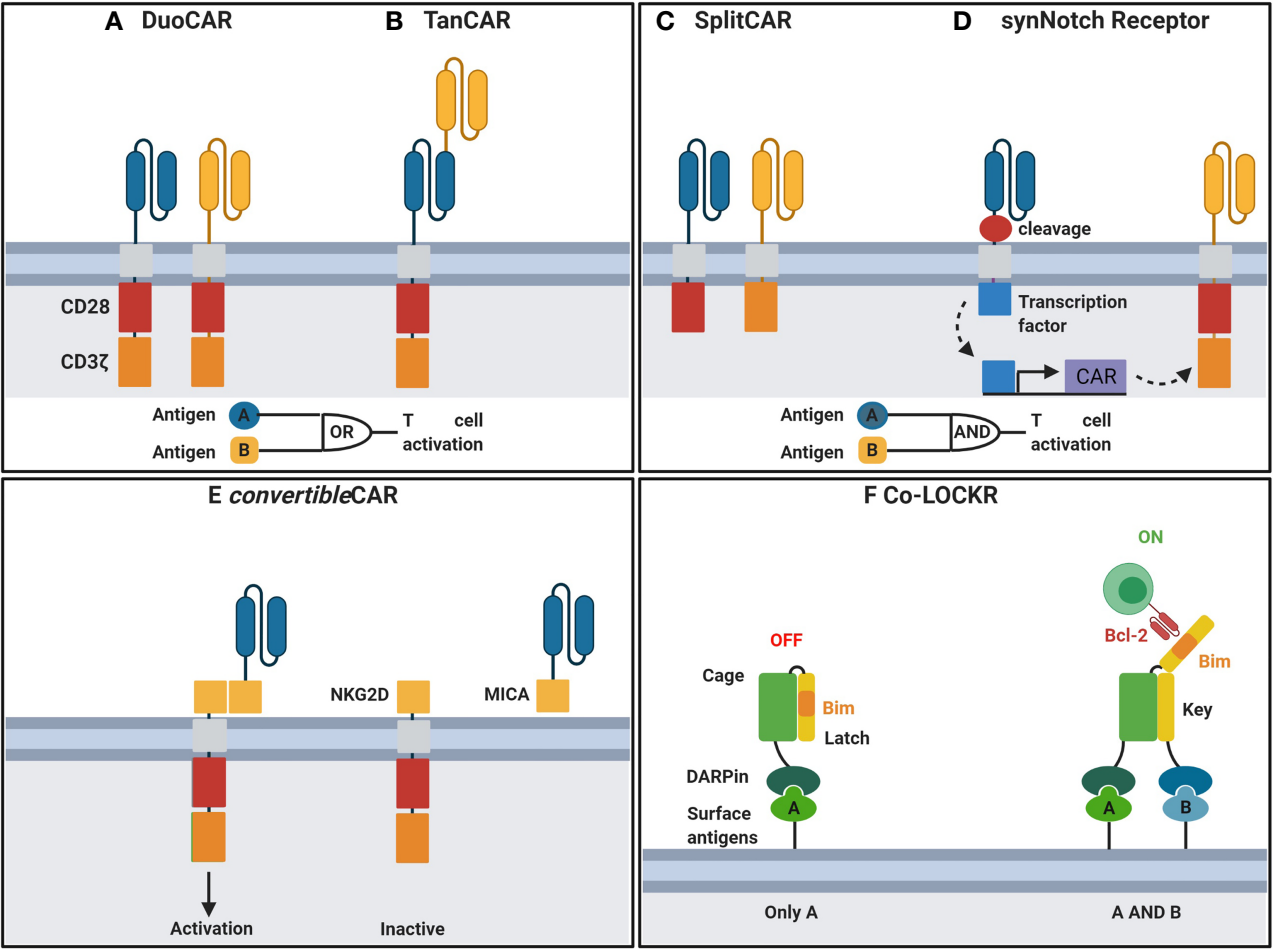
Engineering CAR-T cells for improved function. **(A)** DuoCARs target independent antigens by CAR co-expression. **(B)** TanCARs adopt a tandem antigen recognition domain to target multiple antigens. **(C)** CD3ζ and costimulatory domains are split between independent antigen recognition domains in SplitCARs for T cell activation upon recognition of both antigens. **(D)** Upon recognition of antigen, synNotch receptors undergo transmembrane cleavage and release of their intracellular transcriptional domain which in turn induces the transcription of a secondary CAR for recognition of a second antigen and T cell activation. **(E)** *convertible*CAR, a universal system where the antigen-targeting domain and the T cell signalling unit are split. Effector cells express CARs incorporating NKG2D, the natural receptor of the MIC/ULBP ligand family. MicAbody, comprised of the MICA ligand bound to the scFv of an antibody of interest, is administered separately. **(F)** DARPin linked to a Cage protein bind to surface antigens. Upon binding of a secondary DARPin linked to a key protein to its antigen, the latch is released exposing Bim to bind to a Bcl-2 CAR. CAR, chimeric antigen receptor; NKG2D, MICA receptor; MICA, NKG2D ligand; DARPin, designer ankyrin repeat proteins. Created with BioRender. Adapted from Guedan et al. (105).

Antigen escape, including antigen loss or downregulation, is a major limitation of CARs designed with single antigen recognition domains, particularly for scFv-based CARs. Immune escape was demonstrated in recent clinical trials where a decrease in plasma viremia followed by viral rebound using monotherapy of bNAb VRC01 (103) was observed. In contrast, treatment with combinations of two or more bNAbs significantly reduced the viral reservoir and demonstrated long-term viral suppression (104). Therefore, single bnAb scFv-based CARs may be insufficient for long term HIV suppression due to the emergence of escape mutants. Thus, combination of multiple antigen recognition domain by CARs, using duo or Tandem designs may provide long-term suppression and are currently under investigation. Duo CARs expressing complimentary antigen recognition domains directed against different antigens *via* transfection as a single bicistronic vector (**Figure 2A**) have been used successfully in cancer to overcome immune escape (106). Tandem CAR-T cells consists of a CAR where distinct antigen recognition domains are fused to generate smaller transgenes compared to dual CARs whilst retaining capacity to target multiple antigens can prevent immune escape (**Figure 2B**) (107). More recently, Hajduczki et al. further improved a bispecific CAR design based on the CRD of human mannose-binding lectin (MBL) recognizing the highly conserved oligomannose patch on gp120 and CD4 ligand by addition of a third antigen recognition domain against a distinctly conserved region on Env. The group employed a polypeptide sequence derived from the N-terminus of the HIV coreceptor CCR5 to develop the trispecific CAR to demonstrate enhanced *in vitro* anti-HIV potency compared to the bispecific CAR (108). Binding of either CAR in dual or tandem systems to its associated antigen is sufficient to stimulate full T cell activation. Moreover, cancer studies have demonstrated that enhanced T-cell function is observed when both targets are present (106). A preliminary study of anti-HIV dual CARs designed with CD4 antigen recognition domains linked to bnAb 17b scFvs or carbohydrate recognition domains of a human C-type lectin (109) transduced into CD8 T cells showed enhanced potency against genetically diverse strains of HIV when compared to single CD4 antigen recognition domain-based CARs *in vitro.* Furthermore, Liu et al. demonstrated that bispecific CAR function is influenced by the length between various antigen-binding domains to inform future studies incorporating dual antigen recognition domain designs (110). Subsequent studies have included CD4-based duo CARs linked to various bnAb scFvs or the carbohydrate recognition domain of a human C-type lectin receptor and have all shown long -term suppression of HIV *in vitro* (109, 111). Most recently, a study describes duo CARs targeting two or three antigens simultaneously *via* HIV neutralising antibody fragments in transduced primary human T cells *in vitro* (111). Multi-specific CAR - T cells were able to eliminate PBMCs infected with single bnAb-resistant HIV strains and displayed long-term control of HIV infection *in vivo* and the prevention of CD4 T cell loss (111). Further, the group demonstrated long-term control of HIV infection *in vivo* and prevented the loss of CD4+ T cells during HIV infection using a humanized NSG mouse model (111). Using a bicistronic lentiviral vector to allow for simultaneous expression of both CARs, the group targeted dual antigens on HIV env, first the mD1.22 domain to its CD4 binding site, and subsequently the m36.4 domain to its conformationally exposed binding site (111). Collectively, these data demonstrate that dual-targeting antigen recognition domains are important in overcoming immune escape in the context of an anti-HIV immunotherapy.

#### Overcoming Off-Target Toxicitites

HIV specific CAR-T cells have demonstrated long-term safety in the clinical setting (87–89). However, the primary concern with CD4-ligand based anti-HIV CAR-T cell therapies is the capacity for CD4-MHC II interactions resulting in non-specific T cell activation. Fortunately, cytolysis of MHC II-expressing cells has not been demonstrated in CD4-based anti-HIV CAR-T cell studies (91, 110). Development of anti-HIV bnAb-based CAR T cell therapies specifically targeting the HIV envelope glycoproteins is safer than targeting the CD4 binding partners. It is well established that the non-covalent interaction of the gp120 and gp41 envelope glycoproteins of HIV is disrupted by soluble CD4 binding, resulting in irreversible gp120 release (112). Since CAR-T cell recognition of its specific antigen is an MHC I independent process, another important consideration is the recognition of cell-free or virion-associated HIV env by HIV specific CAR-T cells leading to activation and potentially lethal cytokine release. Although this area has not been extensively explored in HIV CAR-T cell therapies, dual targeting of HIV env with cell surface markers may provide a novel strategy to mitigate off-target cytotoxicity resulting from non-specific T cell responses to cell-free or virion-associated HIV env.

However, targeting two antigens may lead to increased risk for off-target toxicity following CAR-T cell treatment. Strategies to mitigate off-target cytotoxicity include synNotch, LOCKR and convertible systems where CARs are activated upon recognition of a specific combination of antigens. The primary signal can be provided following stimulation of the CAR linked to the CD3ζ chain whilst co-stimulation is split and provided by another CAR to limit non-specific activation (**Figure 2C**) (113, 114). A more recent strategy employs a two-step process where activation of the first receptor known as the synthetic Notch (synNotch) induces the expression of a secondary antigen specific receptor (**Figure 2D**) (115). This AND-gate recognition system ensures T cell activation only occurs when both antigens are expressed on target cells thus enabling precision killing and sparing cells expressing either of the single antigens in isolation. Other AND-gating strategies involve dimerising protein switches, which can serve as lock and keys that are activated only upon the two independent antigens (lock-bound and key-bound) present together. An interesting example of this is described in Lajoie et al. (116) (**Figure 2F**).

Another strategy to reduce toxicity is by allowing for controlled expression of CAR T cells. For example, CARs are directed against an inert molecule which is conjugated to an antigen binding antibody. By infusing various antibodies, the universal CAR can be redirected against various antigens and CAR activity can be controlled by regulating the times and amount of antibody infusions. Most recently Herzig et al. engineered a novel version of universal CAR known as *convertible*CAR (**Figure 2E**) where they combine the cytotoxic T cell with many antibodies (117). This is crucial for controlling HIV as different variants exist and there would be no long-term success with a CAR-T cell carrying a single antibody to fight HIV. In this scenario, Herzig et al. used a ligand from the MIC/ULBP family, expressed on stressed cells, and its receptor NKG2D, expressed on CTL and NK cells. They engineered the *convertible*CAR cell T cell to express NKG2D thus converting them into a potent killer, but only when bound to its partner, a protein called MIC-A with A being any antibody of interest. Consequently, neither the CAR-T cells nor the MicAbody can kill target cells until they have bound to each other and the MicAbody has specifically engaged its epitope on an HIV-infected cell to create an immunologic synapse. This strategy compensated for the cleavage of soluble ligands (MICA and ULBP) for the natural NKG2D receptor from the surface of infected cells, thereby compromising the ability of CTL and NK cells to kill infected cells (118).

The possibility of multiplexing a single *convertible*CAR cell with several MicAbodies makes this platform quite promising for tackling multiple diseases or pathogen variants and for avoiding the universal problem of drug resistance. Another advantage of this type of CAR T cells is the exclusive ligand-receptor interaction where CAR can be introduced into patients in an inert state. Only after the administration of the specific MicAbody or MicAbody mix will the CAR-T get activated. This increases safety and controls dependent on both the dose and timing of administered HIV-specific MicAbody. By contrast, classical CAR T cells are ‘‘on’’ all the time. Using a platform that is inert unless both parts are present can potentially enhance safety.

#### Tissue Microenvironment

A major shared challenge to target cell clearance in chronic HIV infection and cancer is the need for T cell localisation and exertion of effector functions within the immunosuppressive tissue microenvironment. Anti-HIV CAR T cells that over-express the chemokine receptor CXCR5, to promote trafficking into lymphoid tissues have been developed (119). This was based on the identification of follicular CD8 T cells expressing CXCR5 and their control of HIV replication within lymphoid tissue (120), in addition to a proof-of-concept study demonstrating CXCR5 overexpression in CD8 T cells led to localisation within the germinal centres of lymphoid tissue in macaques (121). These CD4-based CXCR5 expressing CAR T cells demonstrated suppression of SIV infection *in vitro* and trafficking in response to CXCL13 in migration assays using transwells and lymph node organoid cultures (122). Most recently, Barber-Anxthelm et al. employed hematopoietic stem cells modified with CD4 ligand based CARs to demonstrate trafficking and multilineage engraftment at lymphoid germinal centres, gut associated lymphoid tissue and the central nervous system (123). Furthermore, CAR expressing cells persisted for nearly two years at these sites of tissue-associated viral reservoirs (123). Since lymph nodes are important sites of HIV reservoirs, these data highlight important innovations that can be included in the development of a dual-targeting HIV-specific CAR-T cell with increased capacities to traffic to anatomical sites that contain large fractions of the latent HIV reservoir. Both the solid tumour microenvironment and lymph nodes can be characterised by the secretion of various cytokines and growth factors by stromal and immune cells to remodel the extracellular matrix of these tissues and contribute to the suppression of T cell responses. To overcome such barriers, CAR T cells have been engineered to secrete extracellular matrix modifying enzymes such as heparinase to degrade heparin sulphate proteoglycans in the extracellular matrix (124). These CAR-T cells demonstrated an improved capacity to infiltrate xenograft tumours in mice and prolonged survival compared with CAR T cells lacking heparinase expression (124). Such strategies could be included in future HIV CAR designs.

After overcoming the physical barriers and localising to the tumour microenvironment, upregulation of inhibitory ligands such as PDL-1 that bind to inhibitory receptor PD1 to suppress effector T cell responses have been observed (125). Several approaches to overcome the effect of PD-1 using CAR T cells have been reported and are succinctly described in Yoon et al. (126). Engineered CAR-T cells co-transduced with truncated PD-1 receptors lacking intracellular signalling functions which still bind PDL-1 but are unable to propagate inhibitory signals have been developed and demonstrated resistance to T cell exhaustion mediated by PDL-1 and prolonged survival in mice bearing xenograft tumours, compared with CAR T cells lacking these truncated PD-1 receptors (127). More recently, Jiang et al. demonstrated that expression of PD-1 dominant negative receptor (DNR) in a 3BNC117 bnAb scFv based CAR T cell resulted in superior lytic and functional responses *in vitro* and *in vivo* when compared to 3BNC117 bnAb scFv CAR expression alone (128). PD-1 receptor expression has also been disrupted completely *via* CRISPR/Cas9 resulting in augmentation of CAR T cell mediated killing of tumor cells *in vitro* and enhanced clearance of PDL-1 expressing tumor xenografts *in vivo* (129). The complexity of the tissue microenvironment continues to drive novel CAR design as previously outlined by our group (130).

#### Cytokine Release Syndrome

The risk of cytokine release syndrome (CRS) is most acute during the first infusion when the cancer tumour burden is at its peak (131). However, the number of HIV-infected target cells in patients on ART is dramatically less than target cell numbers in individuals with leukaemia and HIV reactivation of latently infected cells is characterised by low surface antigen density (132). Tumour specific antigens provoke a potent immune response relative to reactivated latently infected resting memory T cells. For this reason, CRS has not been extensively studied in the context of HIV specific CAR – T cells *in vivo*. However, should life threatening CRS ensue, it would be beneficial to incorporate safety switches to “turn off” the CAR T cells. The popular switches currently being trialled are based on inducible caspase-9 (133) and truncated epidermal growth factor receptor (tEGFR) (elimination induced by the infusion of clinically approved antibody Cetuximab) (134).

#### Expansion and Persistence

The low number of HIV-infected target cells comprising the latent HIV reservoir and their low antigen density upon reactivation leads to challenges surrounding the expansion and persistence of HIV specific CAR – T cells. One strategy involving orthogonal IL-2 cytokine-receptor pairs may overcome poor engineered T cell expansion and persistence upon patient infusion (135). It is known that transcription factor STAT5 is phosphorylated upon IL-2 engagement of its receptor (136). After translocation to the nucleus STAT5 promotes T cell proliferation and cell cycle progression. By engineering orthogonal IL-2 cytokine-receptors pairs, Sockolosky et al. demonstrated that orthogonal IL-2 potently activated STAT5 on orthogonal IL-2 receptor transduced primary mouse CAR – T cells when compared to wild-type resulting in specific expansion of primary mouse CAR – T cells with negligible toxicity whilst retaining anti-tumour effector functions (135). As mentioned previously, 41BB co-stimulated CARs induce a delayed T cell effector response and increased persistence whilst CD28 co-stimulated CARs lead to quicker T cell activation, proliferation, cytolysis. Dual-CARs incorporating distinct CD28 and 4-1BB costimulatory domains can combine both antigen-driven expansion and long-term persistence with potent effector functions (101). Using LRAs to stimulate robust reactivation of latent reservoirs, may help in antigen dependent expansion and persistence of HIV-specific CAR T cells during clinical translation of HIV specific T cell therapies. Thus, a combinatorial approach of LRA and CAR T cells can be expected to aid in increased *in vivo* expansion and persistence.

#### Susceptibility to HIV Infection

CD4 ligand-based HIV specific CARs have illustrated significant barriers to HIV cure due to their susceptibility to HIV infection (137). To overcome this limitation, one strategy describes co-expressing short hairpin RNA (shRNA) sequences targeting CCR5 and HIV LTR to silence target genes (138). It was shown that expressing CCR5 and HIV LTR shRNA decreased the risk of CAR T cell infection leading to overall increased levels and persistence whilst maintaining HIV specific effector functions *in vitro* (138). Targeted CAR integration at the CCR5 locus in primary human T cells has been also demonstrated by RNA-based nuclease expression coupled with adeno-associated virus-mediated delivery of a CCR5-targeting donor template and was shown to effectively supress viral replication in primary HIV infected human T cells (99). Thus, targeted integration of the CAR into the CCR5 locus is an important milestone in developing robust T cell therapies against HIV. More recently, Anthony-Gonda et al. showed infection resistance when the C46 viral fusion inhibitor peptide was co-expressed in anti- HIV CAR T cells without interfering with their effector function (111). Various studies have shown that anti-HIV bnAbs may be used as an alternative to CD4 antigen recognition domains in HIV specific CARs to stimulate specific T cell activation and killing of HIV infected cells without HIV infection of CAR T cells (99, 102, 111). These studies demonstrate the feasibility of utilising bnAb scFv in CAR constructs as immunotherapies for HIV infection.

## Conclusions

Immunotherapy for the treatment of infection and malignancy has revolutionised therapeutic options. While one major aspect of HIV research focusses on developing alternative or complementary methods to LRAs to reactivate HIV, the other exciting aspect focusses on harnessing the immune system more effectively to eliminate HIV reactivated cells. However, none of the attempts at reducing latently infected cells have yet succeeded as CD8 T cells are dysfunctional in chronic HIV infection and LRAs have been suboptimal so far. Many LRAs are toxic, can reactivate HIV but fail to kill the target CD4 T cells, and some inhibit endogenous CD8 T cell function. Currently, there is no licensed treatment for the reduction of the latent HIV reservoir and novel approaches are required to tackle latency. Individual strategies are unikely to eliminate the entire HIV reservoir. However, reactivation *via* multiple approaches such as LRAs or IFN combined with immunotherapy using CD8 CAR T cells to kill reactivated cells need to be tested further. CAR T cells are one such promising immunotherapeutic approach for killing HIV infected cells. An important feature of CD8 CAR T cell immunotherapy is that HIV infected cells that evade CD8 T recognition *via* mutations in MHC-I restricted epitopes or downregulation of MHC-I will be targeted by CAR T cells which recognise surface antigens independent of MHC-I presentation. IFN has also many advantages over classical LRAs: it does not impair the CD8 antiviral function, prevents HIV spread, upregulate MHC-I and is not toxic. In addition to the inclusion of scFvs from bnAbs to induce CAR T cells cytotoxic anti-HIV effects, one could also consider arming the CAR T cells to secrete cytokines that activate latently infected cells and to increase their ability to migrate to key sites of HIV infection such as lymph nodes or mucosal lymphoid tissue or even to immune privileged sites such as the brain *via* the expression of specific homing markers. First generation CAR-T cells were not capable of reducing viral burden permanently in most clinical studies while third and fourth generation led to activation-induced cell death (AICD). However, second generation CAR T cells are currently the most promising candidates as they show superior expansion and reduced HIV infected cells in many *in vitro* and humanised mouse studies. As CAR designs are constantly evolving, their modifications may provide more effective cell therapies to cure HIV infected patients in the future.

## Author Contributions

JY primarily wrote the review under the guidance of KG, AC and NN. SP and KM provided intellectual input. All authors contributed to the article and approved the submitted version.

## Funding

The Westmead Institute for Medical Research paid for open access publication. JY was supported by a grant from the Australian Centre for HIV and Hepatitis Virology Research (ACH2) granted to AC, KM, NN and KG. SP was supported by the Delaney AIDS Research Enterprise (DARE) to Find a Cure funded by the US National Institutes of Health (1UM1AI164560-01) and the Australian National Health and Medical Research Council (APP1149990).

## Conflict of Interest

The authors declare that the research was conducted in the absence of any commercial or financial relationships that could be construed as a potential conflict of interest.

## Publisher’s Note

All claims expressed in this article are solely those of the authors and do not necessarily represent those of their affiliated organizations, or those of the publisher, the editors and the reviewers. Any product that may be evaluated in this article, or claim that may be made by its manufacturer, is not guaranteed or endorsed by the publisher.

## References

[1] Barré-Sinoussi F Ross AL Delfraissy J-F. Past, Present and Future: 30 Years of HIV Research. Nat Rev Microbiol (2013) 11:877–83. doi: 10.1038/nrmicro3132 24162027

[2] Arts EJ Hazuda DJ. HIV-1 Antiretroviral Drug Therapy. Cold Spring Harb Perspect Med (2012) 2:1–23. doi: 10.1101/cshperspect.a007161 PMC3312400 22474613

[3] Sáez-Cirión A Bacchus C Hocqueloux L Avettand-Fenoel V Girault I Lecuroux C. Post-Treatment HIV-1 Controllers With a Long-Term Virological Remission After the Interruption of Early Initiated Antiretroviral Therapy ANRS VISCONTI Study. PloS Pathog (2013) 9:e1003211. doi: 10.1371/journal.ppat.1003211 23516360 PMC3597518

[4] Samri A Bacchus-Souffan C Hocqueloux L Avettand-Fenoel V Descours B Theodorou I. Polyfunctional HIV-Specific T Cells in Post-Treatment Controllers. AIDS (2016) 30:2299–302. doi: 10.1097/QAD.0000000000001195 27428742

[5] Deeken JF Tjen-A-Looi A Rudek MA Okuliar C Young M Little RF. The Rising Challenge of Non-AIDS-Defining Cancers in HIV-Infected Patients. Clin Infect Dis (2012) 55:1228–35. doi: 10.1093/cid/cis613 PMC3529613 22776851

[6] Sonti S Sharma AL Tyagi M. HIV-1 Persistence in the CNS: Mechanisms of Latency, Pathogenesis and an Update on Eradication Strategies. Virus Res (2021) 303:198523. doi: 10.1016/j.virusres.2021.198523 34314771 PMC8966056

[7] O’Neil TR Hu K Truong NR Arshad S Shacklett BL Cunningham AL. The Role of Tissue Resident Memory CD4 T Cells in Herpes Simplex Viral and HIV Infection. Viruses (2021) 13:359. doi: 10.3390/v13030359 33668777 PMC7996247

[8] Deeks SG Archin N Cannon P Collins S Jones RB de Jong MAWP. Research Priorities for an HIV Cure: International AIDS Society Global Scientific Strategy 2021. Nat Med (2021) 27:2085–98. doi: 10.1038/s41591-021-01590-5 34848888

[9] Duette G Hiener B Morgan H Mazur FG Mathivanan V Horsburgh BA. The HIV-1 Proviral Landscape Reveals Nef Contributes to HIV-1 Persistence in Effector Memory CD4+ T-Cells. J Clin Invest (2022) 132:1–17. doi: 10.1172/JCI154422 PMC8970682 35133986

[10] Murray AJ Kwon KJ Farber DL Siliciano RF. The Latent Reservoir for HIV-1: How Immunologic Memory and Clonal Expansion Contribute to HIV-1 Persistence. J Immunol (2016) 197:407–17. doi: 10.4049/jimmunol.1600343 PMC4936486 27382129

[11] McNab F Mayer-Barber K Sher A Wack A O’Garra A. Type I Interferons in Infectious Disease. Nat Rev Immunol (2015) 15:87–103. doi: 10.1038/nri3787 25614319 PMC7162685

[12] Pestka S Krause CD Walter MR. Interferons, Interferon-Like Cytokines, and Their Receptors. Immunol Rev (2004) 202:8–32. doi: 10.1111/j.0105-2896.2004.00204.x 15546383

[13] Witte K Witte E Sabat R Wolk K. IL-28a, IL-28B, and IL-29: Promising Cytokines With Type I Interferon-Like Properties. Cytokine Growth Factor Rev (2010) 21:237–51. doi: 10.1016/j.cytogfr.2010.04.002 20655797

[14] Bertram KM Botting RA Baharlou H Rhodes JW Rana H Graham JD. Identification of HIV Transmitting CD11c + Human Epidermal Dendritic Cells. Nat Commun (2019) 10:2759. doi: 10.1038/s41467-019-10697-w 31227717 PMC6588576

[15] Nasr N Maddocks S Turville SG Harman AN Woolger N Helbig KJ. HIV-1 Infection of Human Macrophages Directly Induces Viperin Which Inhibits Viral Production. Blood (2012) 120:778–88. doi: 10.1182/blood-2012-01-407395 22677126

[16] Harman AN Nasr N Feetham A Galoyan A Alshehri AA Rambukwelle D. HIV Blocks Interferon Induction in Human Dendritic Cells and Macrophages by Dysregulation of TBK1. J Virol (2015) 89:6575–84. doi: 10.1128/JVI.00889-15 PMC4468486 25855743

[17] Doehle BP Chang K Rustagi A McNevin J McElrath MJ Gale M. Vpu Mediates Depletion of Interferon Regulatory Factor 3 During HIV Infection by a Lysosome-Dependent Mechanism. J Virol (2012) 86:8367–74. doi: 10.1128/JVI.00423-12 PMC3421752 22593165

[18] Stacey AR Norris PJ Qin L Haygreen EA Taylor E Heitman J. Induction of a Striking Systemic Cytokine Cascade Prior to Peak Viremia in Acute Human Immunodeficiency Virus Type 1 Infection, in Contrast to More Modest and Delayed Responses in Acute Hepatitis B and C Virus Infections. J Virol (2009) 83:3719–33. doi: 10.1128/JVI.01844-08 PMC2663284 19176632

[19] Sandler NG Bosinger SE Estes JD Zhu RTR Tharp GK Boritz E. Type I Interferon Responses in Rhesus Macaques Prevent SIV Infection and Slow Disease Progression. Nature (2014) 511:601–5. doi: 10.1038/nature13554 PMC4418221 25043006

[20] Li Q Estes JD Schlievert PM Duan L Brosnahan AJ Southern PJ. Glycerol Monolaurate Prevents Mucosal SIV Transmission. Nature (2009) 458:1034–8. doi: 10.1038/nature07831 PMC2785041 19262509

[21] Kolumam GA Thomas S Thompson LJ Sprent J Murali-Krishna K. Type I Interferons Act Directly on CD8 T Cells to Allow Clonal Expansion and Memory Formation in Response to Viral Infection. J Exp Med (2005) 202:637–50. doi: 10.1084/jem.20050821 PMC2212878 16129706

[22] Mikulak J Oriolo F Zaghi E Di Vito C Mavilio D. Natural Killer Cells in HIV-1 Infection and Therapy. AIDS (2017) 31:2317–30. doi: 10.1097/QAD.0000000000001645 PMC5892189 28926399

[23] Ward J Bonaparte M Sacks J Guterman J Fogli M Mavilio D. HIV Modulates the Expression of Ligands Important in Triggering Natural Killer Cell Cytotoxic Responses on Infected Primary T-Cell Blasts. Blood (2007) 110:1207–14. doi: 10.1182/blood-2006-06-028175 PMC1939902 17513617

[24] Douek DC Brenchley JM Betts MR Ambrozak DR Hill BJ Okamoto Y. HIV Preferentially Infects HIV-Specific CD4+ T Cells. Nature (2002) 417:95–8. doi: 10.1038/417095a 11986671

[25] Oxenius A Price DA Easterbrook PJ O’Callaghan CA Kelleher AD Whelan JA. Early Highly Active Antiretroviral Therapy for Acute HIV-1 Infection Preserves Immune Function of CD8+ and CD4+ T Lymphocytes. Proc Natl Acad Sci USA (2000) 97:3382–7. doi: 10.1073/pnas.97.7.3382 PMC16248 10737796

[26] Sun JC Williams MA Bevan MJ. CD4+ T Cells are Required for the Maintenance, Not Programming, of Memory CD8+ T Cells After Acute Infection. Nat Immunol (2004) 5:927–33. doi: 10.1038/ni1105 PMC2776074 15300249

[27] Takata H Buranapraditkun S Kessing C Fletcher JLK Muir R Tardif V. Delayed Differentiation of Potent Effector CD8+ T Cells Reducing Viremia and Reservoir Seeding in Acute HIV Infection. Sci Transl Med (2017) 9:1–22. doi: 10.1126/scitranslmed.aag1809 PMC5678930 28202771

[28] Sáez-Cirión A Lacabaratz C Lambotte O Versmisse P Urrutia A Boufassa F. HIV Controllers Exhibit Potent CD8 T Cell Capacity to Suppress HIV Infection Ex Vivo and Peculiar Cytotoxic T Lymphocyte Activation Phenotype. PNAS (2007) 104:6776–81. doi: 10.1073/pnas.0611244104 PMC1851664 17428922

[29] Borrow P Lewicki H Wei X Horwitz MS Peffer N Meyers H. Antiviral Pressure Exerted by HIV-L-Specific Cytotoxic T Lymphocytes (CTLs) During Primary Infection Demonstrated by Rapid Selection of CTL Escape Virus. Nat Med (1997) 3:205–11. doi: 10.1038/nm0297-205 9018240

[30] Cartwright EK Spicer L Smith SA Lee D Fast R Paganini S. CD8(+) Lymphocytes Are Required for Maintaining Viral Suppression in SIV-Infected Macaques Treated With Short-Term Antiretroviral Therapy. Immunity (2016) 45:656–68. doi: 10.1016/j.immuni.2016.08.018 PMC5087330 27653601

[31] Liu H Liu L Liu K Bizargity P Hancock WW Visner GA. Reduced Cytotoxic Function of Effector CD8+ T Cells Is Responsible for Indoleamine 2,3-Dioxygenase-Dependent Immune Suppression. J Immunol (2009) 183:1022–31. doi: 10.4049/jimmunol.0900408 19564344

[32] Jin X Bauer DE Tuttleton SE Lewin S Gettie A Blanchard J. Dramatic Rise in Plasma Viremia After CD8(+) T Cell Depletion in Simian Immunodeficiency Virus-Infected Macaques. J Exp Med (1999) 189:991–8. doi: 10.1084/jem.189.6.991 PMC2193038 10075982

[33] Leslie AJ Pfafferott KJ Chetty P Draenert R Addo MM Feeney M. HIV Evolution: CTL Escape Mutation and Reversion After Transmission. Nat Med (2004) 10:282–9. doi: 10.1038/nm992 14770175

[34] Turnbull EL Wong M Wang S Wei X Jones NA Conrod KE. Kinetics of Expansion of Epitope-Specific T Cell Responses During Primary HIV-1 Infection. J Immunol (2009) 182:7131–45. doi: 10.4049/jimmunol.0803658 19454710

[35] Ferrari G Korber B Goonetilleke N Liu MKP Turnbull EL Salazar-Gonzalez JF. Relationship Between Functional Profile of HIV-1 Specific CD8 T Cells and Epitope Variability With the Selection of Escape Mutants in Acute HIV-1 Infection. PloS Pathog (2011) 7:e1001273. doi: 10.1371/journal.ppat.1001273 21347345 PMC3037354

[36] Roberts ER Carnathan DG Li H Shaw GM Silvestri G Betts MR. Collapse of Cytolytic Potential in SIV-Specific CD8+ T Cells Following Acute SIV Infection in Rhesus Macaques. PloS Pathog (2016) 12:1–21. doi: 10.1371/journal.ppat.1006135 PMC5231392 28036372

[37] Helleberg M Kronborg G Ullum H Ryder LP Obel N Gerstoft J. Course and Clinical Significance of CD8+ T-Cell Counts in a Large Cohort of HIV-Infected Individuals. J Infect Dis (2015) 211:1726–34. doi: 10.1093/infdis/jiu669 25489001

[38] Perdomo-Celis F Taborda NA Rugeles MT. CD8+ T-Cell Response to HIV Infection in the Era of Antiretroviral Therapy. Front Immunol (2019) 10:1896. doi: 10.3389/fimmu.2019.01896 31447862 PMC6697065

[39] Petrovas C Casazza JP Brenchley JM Price DA Gostick E Adams WC. PD-1 is a Regulator of Virus-Specific CD8+ T Cell Survival in HIV Infection. J Exp Med (2006) 203:2281–92. doi: 10.1084/jem.20061496 PMC2118095 16954372

[40] Dornadula G Zhang H VanUitert B Stern J Livornese L Ingerman MJ. Residual HIV-1 RNA in Blood Plasma of Patients Taking Suppressive Highly Active Antiretroviral Therapy. JAMA (1999) 282:1627–32. doi: 10.1001/jama.282.17.1627 10553788

[41] Schweneker M Favre D Martin JN Deeks SG McCune JM. HIV-Induced Changes in T Cell Signaling Pathways. J Immunol (2008) 180:6490–500. doi: 10.4049/jimmunol.180.10.6490 PMC2648824 18453567

[42] Wherry EJ Ha S-J Kaech SM Haining WN Sarkar S Kalia V. Molecular Signature of CD8+ T Cell Exhaustion During Chronic Viral Infection. Immunity (2007) 27:670–84. doi: 10.1016/j.immuni.2007.09.006 17950003

[43] Palella FJ Baker RK Moorman AC Chmiel JS Wood KC Brooks JT. HIV Outpatient Study Investigators. Mortality in the Highly Active Antiretroviral Therapy Era: Changing Causes of Death and Disease in the HIV Outpatient Study. J Acquir Immune Defic Syndr (2006) 43:27–34. doi: 10.1097/01.qai.0000233310.90484.16 16878047

[44] Vansant G Bruggemans A Janssens J Debyser Z. Block-And-Lock Strategies to Cure HIV Infection. Viruses (2020) 12:E84. doi: 10.3390/v12010084 31936859 PMC7019976

[45] Ahlenstiel CL Suzuki K Marks K Symonds GP Kelleher AD. Controlling HIV-1: Non-Coding RNA Gene Therapy Approaches to a Functional Cure. Front Immunol (2015) 6:474. doi: 10.3389/fimmu.2015.00474 26441979 PMC4584958

[46] Boukli NM Shetty V Cubano L Ricaurte M Coelho-Dos-Reis J Nickens Z. Unique and Differential Protein Signatures Within the Mononuclear Cells of HIV-1 and HCV Mono-Infected and Co-Infected Patients. Clin Proteomics (2012) 9:11. doi: 10.1186/1559-0275-9-11 22958358 PMC3582525

[47] Vozzolo L Loh B Gane PJ Tribak M Zhou L Anderson I. Gyrase B Inhibitor Impairs HIV-1 Replication by Targeting Hsp90 and the Capsid Protein. J Biol Chem (2010) 285:39314–28. doi: 10.1074/jbc.M110.155275 PMC2998086 20937817

[48] Rice AP. The HIV-1 Tat Protein: Mechanism of Action and Target for HIV-1 Cure Strategies. Curr Pharm Des (2017) 23:4098–102. doi: 10.2174/1381612823666170704130635 PMC5700838 28677507

[49] Kessing CF Nixon CC Li C Tsai P Takata H Mousseau G. In Vivo Suppression of HIV Rebound by Didehydro-Cortistatin A, a “Block-And-Lock” Strategy for HIV-1 Treatment. Cell Rep (2017) 21:600–11. doi: 10.1016/j.celrep.2017.09.080 PMC5653276 29045830

[50] Xiao Q Guo D Chen S. Application of CRISPR/Cas9-Based Gene Editing in HIV-1/AIDS Therapy. Front Cell Infect Microbiol (2019) 9:69. doi: 10.3389/fcimb.2019.00069 30968001 PMC6439341

[51] Thorlund K Horwitz MS Fife BT Lester R Cameron DW. Landscape Review of Current HIV ‘Kick and Kill’ Cure Research - Some Kicking, Not Enough Killing. BMC Infect Dis (2017) 17:595. doi: 10.1186/s12879-017-2683-3 28851294 PMC5576299

[52] Rasmussen TA Tolstrup M Søgaard OS. Reversal of Latency as Part of a Cure for HIV-1. Trends Microbiol (2016) 24:90–7. doi: 10.1016/j.tim.2015.11.003 26690612

[53] Marsden MD Wu X Navab SM Loy BA Schrier AJ DeChristopher BA. Characterization of Designed, Synthetically Accessible Bryostatin Analog HIV Latency Reversing Agents. Virology (2018) 520:83–93. doi: 10.1016/j.virol.2018.05.006 29800728 PMC6018613

[54] Pache L Dutra MS Spivak AM Marlett JM Murry JP Hwang Y. BIRC2/cIAP1 Is a Negative Regulator of HIV-1 Transcription and Can Be Targeted by Smac Mimetics to Promote Reversal of Viral Latency. Cell Host Microbe (2015) 18:345–53. doi: 10.1016/j.chom.2015.08.009 PMC4617541 26355217

[55] Kim Y Anderson JL Lewin SR. Getting the “Kill” Into “Shock and Kill”: Strategies to Eliminate Latent HIV. Cell Host Microbe (2018) 23:14–26. doi: 10.1016/j.chom.2017.12.004 29324227 PMC5990418

[56] Walker-Sperling VE Pohlmeyer CW Tarwater PM Blankson JN. The Effect of Latency Reversal Agents on Primary CD8+ T Cells: Implications for Shock and Kill Strategies for Human Immunodeficiency Virus Eradication. EBioMedicine (2016) 8:217–29. doi: 10.1016/j.ebiom.2016.04.019 PMC4919475 27428432

[57] Tsai A Irrinki A Kaur J Cihlar T Kukolj G Sloan DD. Toll-Like Receptor 7 Agonist GS-9620 Induces HIV Expression and HIV-Specific Immunity in Cells From HIV-Infected Individuals on Suppressive Antiretroviral Therapy. J Virol (2017) 91:e02166-16. doi: 10.1128/JVI.02166-16 28179531 PMC5375698

[58] Petravic J Rasmussen TA Lewin SR Kent SJ Davenport MP. Relationship Between Measures of HIV Reactivation and Decline of the Latent Reservoir Under Latency-Reversing Agents. J Virol (2017) 91:1–14. doi: 10.1128/JVI.02092-16 PMC5391444 28202759

[59] Sluis RMV d Kumar NA Pascoe RD Zerbato JM Evans VA Dantanarayana AI. Combination Immune Checkpoint Blockade to Reverse HIV Latency. J Immunol (2020) 204(5):1242–54. doi: 10.4049/jimmunol.1901191 PMC7354848 31988180

[60] Tong O Duette G O’Neil TR Royle CM Rana H Johnson B. Plasmacytoid Dendritic Cells Have Divergent Effects on HIV Infection of Initial Target Cells and Induce a Pro-Retention Phenotype. PloS Pathog (2021) 17:e1009522. doi: 10.1371/journal.ppat.1009522 33872331 PMC8084337

[61] Lavender KJ Gibbert K Peterson KE Dis EV Francois S Woods T. Interferon Alpha Subtype-Specific Suppression of HIV-1 Infection *In Vivo* . J Virol (2016) 90:6001–13. doi: 10.1128/JVI.00451-16 PMC4907223 27099312

[62] Schröder ARW Shinn P Chen H Berry C Ecker JR Bushman F. HIV-1 Integration in the Human Genome Favors Active Genes and Local Hotspots. Cell (2002) 110:521–9. doi: 10.1016/s0092-8674(02)00864-4 12202041

[63] Jones RB Mueller S O’Connor R Rimpel K Sloan DD Karel D. A Subset of Latency-Reversing Agents Expose HIV-Infected Resting CD4+ T-Cells to Recognition by Cytotoxic T-Lymphocytes. PloS Pathog (2016) 12:e1005545. doi: 10.1371/journal.ppat.1005545 27082643 PMC4833318

[64] Sadowski I Hashemi FB. Strategies to Eradicate HIV From Infected Patients: Elimination of Latent Provirus Reservoirs. Cell Mol Life Sci (2019) 76:3583–600. doi: 10.1007/s00018-019-03156-8 PMC6697715 31129856

[65] Huang S-H Ren Y Thomas AS Chan D Mueller S Ward AR. Latent HIV Reservoirs Exhibit Inherent Resistance to Elimination by CD8^+^ T Cells. J Clin Invest (2018) 128:876–89. doi: 10.1172/JCI97555 PMC5785246 29355843

[66] Garrido C Abad-Fernandez M Tuyishime M Pollara JJ Ferrari G Soriano-Sarabia N. Interleukin-15-Stimulated Natural Killer Cells Clear HIV-1-Infected Cells Following Latency Reversal Ex Vivo. J Virol (2018) 92:1–13. doi: 10.1128/JVI.00235-18 PMC5974478 29593039

[67] Chandrasekar AP Cummins NW Badley AD. The Role of the BCL-2 Family of Proteins in HIV-1 Pathogenesis and Persistence. Clin Microbiol Rev (2019) 33:1–25. doi: 10.1128/CMR.00107-19 PMC6822993 31666279

[68] Ren Y Huang SH Patel S Alberto WDC Magat D Ahimovic D. BCL-2 Antagonism Sensitizes Cytotoxic T Cell–Resistant HIV Reservoirs to Elimination Ex Vivo. J Clin Invest (2020) 130:2542–59. doi: 10.1172/JCI132374 PMC7191002 32027622

[69] Tomaras GD Yates NL Liu P Qin L Fouda GG Chavez LL. Initial B-Cell Responses to Transmitted Human Immunodeficiency Virus Type 1: Virion-Binding Immunoglobulin M (IgM) and IgG Antibodies Followed by Plasma Anti-Gp41 Antibodies With Ineffective Control of Initial Viremia. J Virol (2008) 82:12449–63. doi: 10.1128/JVI.01708-08 PMC2593361 18842730

[70] Bonsignori M Liao H-X Gao F Williams WB Alam SM Montefiori DC. Antibody-Virus Co-Evolution in HIV Infection: Paths for HIV Vaccine Development. Immunol Rev (2017) 275:145–60. doi: 10.1111/imr.12509 PMC5302796 28133802

[71] Jacobson JM Colman N Ostrow NA Simson RW Tomesch D Marlin L. Passive Immunotherapy in the Treatment of Advanced Human Immunodeficiency Virus Infection. J Infect Dis (1993) 168:298–305. doi: 10.1093/infdis/168.2.298 8101550

[72] Barouch DH Whitney JB Moldt B Klein F Oliveira TY Liu J. Therapeutic Efficacy of Potent Neutralizing HIV-1-Specific Monoclonal Antibodies in SHIV-Infected Rhesus Monkeys. Nature (2013) 503:224–8. doi: 10.1038/nature12744 PMC4017780 24172905

[73] Halper-Stromberg A Nussenzweig MC. Towards HIV-1 Remission: Potential Roles for Broadly Neutralizing Antibodies. J Clin Invest (2016) 126:415–23. doi: 10.1172/JCI80561 PMC4731188 26752643

[74] Lal KG Naluyima P Costanzo MC Kijak GH Eller LA Creegan M. Terminally Differentiated CD8 Effector T Cells Have NK-Like Features and are Potent Mediators of HIV-Specific ADCC. J Immunol (2017) 198:125.4–4.

[75] Caskey M. Broadly Neutralizing Antibodies for the Treatment and Prevention of HIV Infection. Curr Opin HIV AIDS (2020) 15:49–55. doi: 10.1097/COH.0000000000000600 31764199 PMC7340121

[76] Gaudinski MR Houser KV Doria-Rose NA Chen GL Rothwell RSS Berkowitz N. Safety and Pharmacokinetics of Broadly Neutralising Human Monoclonal Antibody VRC07-523LS in Healthy Adults: A Phase 1 Dose-Escalation Clinical Trial. Lancet HIV (2019) 6:e667–e679. doi: 10.1016/S2352-3018(19)30181-X 31473167 PMC11100866

[77] Dirk BS Pawlak EN Johnson AL Van Nynatten LR Jacob RA Heit B. HIV-1 Nef Sequesters MHC-I Intracellularly by Targeting Early Stages of Endocytosis and Recycling. Sci Rep (2016) 6:37021. doi: 10.1038/srep37021 27841315 PMC5107982

[78] Rafiq S Hackett CS Brentjens RJ. Engineering Strategies to Overcome the Current Roadblocks in CAR T Cell Therapy. Nat Rev Clin Oncol (2020) 17:147–67. doi: 10.1038/s41571-019-0297-y PMC7223338 31848460

[79] Hamilton JR Tsuchida CA Nguyen DN Shy BR McGarrigle ER Sandoval Espinoza CR. Targeted Delivery of CRISPR-Cas9 and Transgenes Enables Complex Immune Cell Engineering. Cell Rep (2021) 35:109207. doi: 10.1016/j.celrep.2021.109207 34077734 PMC8236216

[80] Bishop DC Xu N Tse B O’Brien TA Gottlieb DJ Dolnikov A. PiggyBac-Engineered T Cells Expressing CD19-Specific CARs That Lack IgG1 Fc Spacers Have Potent Activity Against B-ALL Xenografts. Mol Ther (2018) 26:1883–95. doi: 10.1016/j.ymthe.2018.05.007 PMC6094355 29861327

[81] Beatty GL O’Hara MH Lacey SF Torigian DA Nazimuddin F Chen F. Activity of Mesothelin-Specific Chimeric Antigen Receptor T Cells Against Pancreatic Carcinoma Metastases in a Phase 1 Trial. Gastroenterology (2018) 155:29–32. doi: 10.1053/j.gastro.2018.03.029 29567081 PMC6035088

[82] Jin C Fotaki G Ramachandran M Nilsson B Essand M Yu D. Safe Engineering of CAR T Cells for Adoptive Cell Therapy of Cancer Using Long-Term Episomal Gene Transfer. EMBO Mol Med (2016) 8:702–11. doi: 10.15252/emmm.201505869 PMC4931286 27189167

[83] Roth TL Puig-Saus C Yu R Shifrut E Carnevale J Li PJ. Reprogramming Human T Cell Function and Specificity With non-Viral Genome Targeting. Nature (2018) 559:405–9. doi: 10.1038/s41586-018-0326-5 PMC6239417 29995861

[84] Maude SL Frey N Shaw PA Aplenc R Barrett DM Bunin NJ. Chimeric Antigen Receptor T Cells for Sustained Remissions in Leukemia. N Engl J Med (2014) 371:1507–17. doi: 10.1056/NEJMoa1407222 PMC4267531 25317870

[85] Melenhorst JJ Chen GM Wang M Porter DL Chen C Collins MA. Decade-Long Leukaemia Remissions With Persistence of CD4+ CAR T Cells. Nature (2022) 602:503–9. doi: 10.1038/s41586-021-04390-6 PMC9166916 35110735

[86] Neelapu SS. Managing the Toxicities of CAR T-Cell Therapy. Hematol Oncol (2019) 37(Suppl 1):48–52. doi: 10.1002/hon.2595 31187535

[87] Walker RE Bechtel CM Natarajan V Baseler M Hege KM Metcalf JA. Long-Term *In Vivo* Survival of Receptor-Modified Syngeneic T Cells in Patients With Human Immunodeficiency Virus Infection. Blood (2000) 96:467–74. doi: 10.1182/blood.V96.2.467 10887107

[88] Mitsuyasu RT Anton PA Deeks SG Scadden DT Connick E Downs MT. Prolonged Survival and Tissue Trafficking Following Adoptive Transfer of CD4zeta Gene-Modified Autologous CD4(+) and CD8(+) T Cells in Human Immunodeficiency Virus-Infected Subjects. Blood (2000) 96:785–93. doi: 10.1182/blood.V96.3.785.015k10_785_793 10910888

[89] Deeks SG Wagner B Anton PA Mitsuyasu RT Scadden DT Huang C. A Phase II Randomized Study of HIV-Specific T-Cell Gene Therapy in Subjects With Undetectable Plasma Viremia on Combination Antiretroviral Therapy. Mol Ther (2002) 5:788–97. doi: 10.1006/mthe.2002.0611 12027564

[90] Iwamoto N Patel B Song K Mason R Bolivar-Wagers S Bergamaschi C. Evaluation of Chimeric Antigen Receptor T Cell Therapy in non-Human Primates Infected With SHIV or SIV. PloS One (2021) 16:e0248973. doi: 10.1371/journal.pone.0248973 33752225 PMC7984852

[91] Romeo C Seed B. Cellular Immunity to HIV Activated by CD4 Fused to T Cell or Fc Receptor Polypeptides. Cell (1991) 64:1037–46. doi: 10.1016/0092-8674(91)90327-u 1900456

[92] Roberts M Qin L Zhang D Smith D Tran A Dull T. Targeting of Human Immunodeficiency Virus-Infected Cells by CD8+ T Lymphocytes Armed With Universal T-Cell Receptors. Blood (1994) 84:2878–89. doi: 10.1182/blood.V84.9.2878.2878 7949163

[93] Yang OO Tran A-C Kalams SA Johnson RP Roberts MR Walker BD. Lysis of HIV-1-Infected Cells and Inhibition of Viral Replication by Universal Receptor T Cells. PNAS (1997) 94:11478–83. doi: 10.1073/pnas.94.21.11478 PMC23511 9326635

[94] Leibman RS Richardson MW Ellebrecht CT Maldini CR Glover JA Secreto AJ. Supraphysiologic Control Over HIV-1 Replication Mediated by CD8 T Cells Expressing a Re-Engineered CD4-Based Chimeric Antigen Receptor. PloS Pathog (2017) 13:e1006613. doi: 10.1371/journal.ppat.1006613 29023549 PMC5638568

[95] Kaartinen T Luostarinen A Maliniemi P Keto J Arvas M Belt H. Low Interleukin-2 Concentration Favors Generation of Early Memory T Cells Over Effector Phenotypes During Chimeric Antigen Receptor T-Cell Expansion. Cytotherapy (2017) 19:689–702. doi: 10.1016/j.jcyt.2017.03.067 28411126

[96] Long AH Haso WM Shern JF Wanhainen KM Murgai M Ingaramo M. 4-1BB Costimulation Ameliorates T Cell Exhaustion Induced by Tonic Signaling of Chimeric Antigen Receptors. Nat Med (2015) 21:581–90. doi: 10.1038/nm.3838 PMC4458184 25939063

[97] Zhen A Peterson CW Carrillo MA Reddy SS Youn CS Lam BB. Long-Term Persistence and Function of Hematopoietic Stem Cell-Derived Chimeric Antigen Receptor T Cells in a Nonhuman Primate Model of HIV/AIDS. PloS Pathog (2017) 13:e1006753. doi: 10.1371/journal.ppat.1006753 29284044 PMC5746250

[98] Seif M Einsele H Löffler J. CAR T Cells Beyond Cancer: Hope for Immunomodulatory Therapy of Infectious Diseases. Front Immunol (2019) 10:2711. doi: 10.3389/fimmu.2019.02711 31824500 PMC6881243

[99] Hale M Mesojednik T Ibarra GSR Sahni J Bernard A Sommer K. Engineering HIV-Resistant, Anti-HIV Chimeric Antigen Receptor T Cells. Mol Ther (2017) 25:570–9. doi: 10.1016/j.ymthe.2016.12.023 PMC5363191 28143740

[100] Maldini CR Gayout K Leibman RS Dopkin DL Mills JP Shan X. HIV-Resistant and HIV-Specific CAR-Modified CD4+ T Cells Mitigate HIV Disease Progression and Confer CD4+ T Cell Help *In Vivo* . Mol Ther (2020) 28:1585–99. doi: 10.1016/j.ymthe.2020.05.012 PMC7335752 32454027

[101] Maldini CR Claiborne DT Okawa K Chen T Dopkin DL Shan X. Dual CD4-Based CAR T Cells With Distinct Costimulatory Domains Mitigate HIV Pathogenesis *In Vivo* . Nat Med (2020) 26:1776–87. doi: 10.1038/s41591-020-1039-5 PMC9422086 32868878

[102] Liu B Zou F Lu L Chen C He D Zhang X. Chimeric Antigen Receptor T Cells Guided by the Single-Chain Fv of a Broadly Neutralizing Antibody Specifically and Effectively Eradicate Virus Reactivated From Latency in CD4+ T Lymphocytes Isolated From HIV-1-Infected Individuals Receiving Suppressive Combined Antiretroviral Therapy. J Virol (2016) 90:9712–24. doi: 10.1128/JVI.00852-16 PMC5068523 27535056

[103] Bar KJ Sneller MC Harrison LJ Justement JS Overton ET Petrone ME. Effect of HIV Antibody VRC01 on Viral Rebound After Treatment Interruption. N Engl J Med (2016) 375:2037–50. doi: 10.1056/NEJMoa1608243 PMC5292134 27959728

[104] Mendoza P Gruell H Nogueira L Pai JA Butler AL Millard K. Combination Therapy With Anti-HIV-1 Antibodies Maintains Viral Suppression. Nature (2018) 561:479–84. doi: 10.1038/s41586-018-0531-2 PMC6166473 30258136

[105] Guedan S Calderon H Posey AD Maus MV. Engineering and Design of Chimeric Antigen Receptors. Mol Ther Methods Clin Dev (2019) 12:145–56. doi: 10.1016/j.omtm.2018.12.009 PMC6330382 30666307

[106] Ruella M Barrett DM Kenderian SS Shestova O Hofmann TJ Perazzelli J. Dual CD19 and CD123 Targeting Prevents Antigen-Loss Relapses After CD19-Directed Immunotherapies. J Clin Invest (2016) 126:3814–26. doi: 10.1172/JCI87366 PMC5096828 27571406

[107] Fry TJ Shah NN Orentas RJ Stetler-Stevenson M Yuan CM Ramakrishna S. CD22-Targeted CAR T Cells Induce Remission in B-ALL That is Naive or Resistant to CD19-Targeted CAR Immunotherapy. Nat Med (2018) 24:20–8. doi: 10.1038/nm.4441 PMC5774642 29155426

[108] Hajduczki A Danielson DT Elias DS Bundoc V Scanlan AW Berger EA. A Trispecific Anti-HIV Chimeric Antigen Receptor Containing the CCR5 N-Terminal Region. Front Cell Infect Microbiol (2020) 10:242. doi: 10.3389/fcimb.2020.00242 32523897 PMC7261873

[109] Ghanem MH Bolivar-Wagers S Dey B Hajduczki A Vargas-Inchaustegui DA Danielson DT. Bispecific Chimeric Antigen Receptors Targeting the CD4 Binding Site and High-Mannose Glycans of Gp120 Optimized for Anti-Human Immunodeficiency Virus Potency and Breadth With Minimal Immunogenicity. Cytotherapy (2018) 20:407–19. doi: 10.1016/j.jcyt.2017.11.001 29306566

[110] Liu L Patel B Ghanem MH Bundoc V Zheng Z Morgan RA. Novel CD4-Based Bispecific Chimeric Antigen Receptor Designed for Enhanced Anti-HIV Potency and Absence of HIV Entry Receptor Activity. J Virol (2015) 89:6685–94. doi: 10.1128/JVI.00474-15 PMC4468509 25878112

[111] Anthony-Gonda K Bardhi A Ray A Flerin N Li M Chen W. Multispecific Anti-HIV duoCAR-T Cells Display Broad *In Vitro* Antiviral Activity and Potent *In Vivo* Elimination of HIV-Infected Cells in a Humanized Mouse Model. Sci Transl Med (2019) 11:1–40. doi: 10.1126/scitranslmed.aav5685 PMC7136029 31391322

[112] Moore JP McKeating JA Weiss RA Sattentau QJ. Dissociation of Gp120 From HIV-1 Virions Induced by Soluble CD4. Science (1990) 250:1139–42. doi: 10.1126/science.2251501 2251501

[113] Kloss CC Condomines M Cartellieri M Bachmann M Sadelain M. Combinatorial Antigen Recognition With Balanced Signaling Promotes Selective Tumor Eradication by Engineered T Cells. Nat Biotechnol (2013) 31:71–5. doi: 10.1038/nbt.2459 PMC5505184 23242161

[114] Lanitis E Poussin M Klattenhoff AW Song D Sandaltzopoulos R June CH. Chimeric Antigen Receptor T Cells With Dissociated Signaling Domains Exhibit Focused Antitumor Activity With Reduced Potential for Toxicity *In Vivo* . Cancer Immunol Res (2013) 1:43–53. doi: 10.1158/2326-6066.CIR-13-0008 24409448 PMC3881605

[115] Roybal KT Williams JZ Morsut L Rupp LJ Kolinko I Choe JH. Engineering T Cells With Customized Therapeutic Response Programs Using Synthetic Notch Receptors. Cell (2016) 167:419–432.e16. doi: 10.1016/j.cell.2016.09.011 27693353 PMC5072533

[116] Lajoie MJ Boyken SE Salter AI Bruffey J Rajan A Langan RA. Designed Protein Logic to Target Cells With Precise Combinations of Surface Antigens. Science (2020) 369:1637–43. doi: 10.1126/science.aba6527 PMC8085813 32820060

[117] Herzig E Kim KC Packard TA Vardi N Schwarzer R Gramatica A. Attacking Latent HIV With convertibleCAR-T Cells, a Highly Adaptable Killing Platform. Cell (2019) 179:880–894.e10. doi: 10.1016/j.cell.2019.10.002 31668804 PMC6922308

[118] Matusali G Tchidjou HK Pontrelli G Bernardi S D’Ettorre G Vullo V. Soluble Ligands for the NKG2D Receptor are Released During HIV-1 Infection and Impair NKG2D Expression and Cytotoxicity of NK Cells. FASEB J (2013) 27:2440–50. doi: 10.1096/fj.12-223057 23395909

[119] Haran KP Hajduczki A Pampusch MS Mwakalundwa G Vargas-Inchaustegui DA Rakasz EG. Simian Immunodeficiency Virus (SIV)-Specific Chimeric Antigen Receptor-T Cells Engineered to Target B Cell Follicles and Suppress SIV Replication. Front Immunol (2018) 9:492. doi: 10.3389/fimmu.2018.00492 29616024 PMC5869724

[120] Leong YA Chen Y Ong HS Wu D Man K Deleage C. CXCR5(+) Follicular Cytotoxic T Cells Control Viral Infection in B Cell Follicles. Nat Immunol (2016) 17:1187–96. doi: 10.1038/ni.3543 27487330

[121] Ayala VI Deleage C Trivett MT Jain S Coren LV Breed MW. CXCR5-Dependent Entry of CD8 T Cells Into Rhesus Macaque B-Cell Follicles Achieved Through T-Cell Engineering. J Virol (2017) 91:1–17. doi: 10.1128/JVI.02507-16 PMC5432868 28298605

[122] Pampusch MS Hajduczki A Mwakalundwa G Connick E Berger EA Skinner PJ. Production and Characterization of SIV-Specific CAR/CXCR5 T Cells. In: Rast J Buckley K, editors. Immune Receptors: Methods and Protocols. Methods in Molecular Biology. New York, NY: Springer US (2022). p. 171–85. doi: 10.1007/978-1-0716-1944-5_12 34870819

[123] Barber-Axthelm IM Barber-Axthelm V Sze KY Zhen A Suryawanshi GW Chen ISY. Stem Cell–Derived CAR T Cells Traffic to HIV Reservoirs in Macaques. JCI Insight (2021) 6:e141502. doi: 10.1172/jci.insight.141502 PMC7821595 33427210

[124] Caruana I Savoldo B Hoyos V Weber G Liu H Kim ES. Heparanase Promotes Tumor Infiltration and Antitumor Activity of CAR-Redirected T Lymphocytes. Nat Med (2015) 21:524–9. doi: 10.1038/nm.3833 PMC4425589 25849134

[125] Spranger S Spaapen RM Zha Y Williams J Meng Y Ha TT. Up-Regulation of PD-L1, IDO, and Tregs in the Melanoma Tumor Microenvironment Is Driven by CD8+ T Cells. Sci Trans Med (2013) 5:200ra116–200ra116. doi: 10.1126/scitranslmed.3006504 PMC4136707 23986400

[126] Yoon DH Osborn MJ Tolar J Kim CJ. Incorporation of Immune Checkpoint Blockade Into Chimeric Antigen Receptor T Cells (CAR-Ts): Combination or Built-In CAR-T. Int J Mol Sci (2018) 19:E340. doi: 10.3390/ijms19020340 29364163 PMC5855562

[127] Cherkassky L Morello A Villena-Vargas J Feng Y Dimitrov DS Jones DR. Human CAR T Cells With Cell-Intrinsic PD-1 Checkpoint Blockade Resist Tumor-Mediated Inhibition. J Clin Invest (2016) 126:3130–44. doi: 10.1172/JCI83092 PMC4966328 27454297

[128] Jiang Z Liang H Pan H Liang Y Wang H Yang X. HIV-1-Specific CAR-T Cells With Cell-Intrinsic PD-1 Checkpoint Blockade Enhance Anti-HIV Efficacy *In Vivo* . Front Microbiol (2021) 12:684016. doi: 10.3389/fmicb.2021.684016 34295319 PMC8290485

[129] Rupp LJ Schumann K Roybal KT Gate RE Ye CJ Lim WA. CRISPR/Cas9-Mediated PD-1 Disruption Enhances Anti-Tumor Efficacy of Human Chimeric Antigen Receptor T Cells. Sci Rep (2017) 7:737. doi: 10.1038/s41598-017-00462-8 28389661 PMC5428439

[130] Habib R Nagrial A Micklethwaite K Gowrishankar K. Chimeric Antigen Receptors for the Tumour Microenvironment. In: Birbrair A, editor. Tumor Microenvironment: State of the Science. Advances in Experimental Medicine and Biology. Cham: Springer International Publishing (2020). p. 117–43. doi: 10.1007/978-3-030-44518-8_8 32588326

[131] Shimabukuro-Vornhagen A Gödel P Subklewe M Stemmler HJ Schlößer HA Schlaak M. Cytokine Release Syndrome. J ImmunoTher Cancer (2018) 6:56. doi: 10.1186/s40425-018-0343-9 29907163 PMC6003181

[132] Massanella M Richman DD. Measuring the Latent Reservoir *In Vivo* . J Clin Invest (2016) 126:464–72. doi: 10.1172/JCI80567 PMC4731179 26829625

[133] Zhang P Raju J Ullah MA Au R Varelias A Gartlan KH. Phase I Trial of Inducible Caspase 9 T Cells in Adult Stem Cell Transplant Demonstrates Massive Clonotypic Proliferative Potential and Long-Term Persistence of Transgenic T Cells. Clin Cancer Res (2019) 25:1749–55. doi: 10.1158/1078-0432.CCR-18-3069 30765390

[134] Paszkiewicz PJ Fräßle SP Srivastava S Sommermeyer D Hudecek M Drexler I. Targeted Antibody-Mediated Depletion of Murine CD19 CAR T Cells Permanently Reverses B Cell Aplasia. J Clin Invest (2016) 126:4262–72. doi: 10.1172/JCI84813 PMC5096899 27760047

[135] Sockolosky JT Trotta E Parisi G Picton L Su LL Le AC. Selective Targeting of Engineered T Cells Using Orthogonal IL-2 Cytokine-Receptor Complexes. Science (2018) 359:1037–42. doi: 10.1126/science.aar3246 PMC5947856 29496879

[136] Moriggl R Sexl V Piekorz R Topham D Ihle JN. Stat5 Activation is Uniquely Associated With Cytokine Signaling in Peripheral T Cells. Immunity (1999) 11:225–30. doi: 10.1016/s1074-7613(00)80097-7 10485657

[137] Brenchley JM Schacker TW Ruff LE Price DA Taylor JH Beilman GJ. CD4+ T Cell Depletion During All Stages of HIV Disease Occurs Predominantly in the Gastrointestinal Tract. J Exp Med (2004) 200:749–59. doi: 10.1084/jem.20040874 PMC2211962 15365096

[138] Ringpis G-EE Shimizu S Arokium H Camba-Colón J Carroll MV Cortado R. Engineering HIV-1-Resistant T-Cells From Short-Hairpin RNA-Expressing Hematopoietic Stem/Progenitor Cells in Humanized BLT Mice. PloS One (2012) 7:e53492. doi: 10.1371/journal.pone.0053492 23300932 PMC3534037

